# Obstructive hypertrophic cardiomyopathy: from genetic insights to a multimodal therapeutic approach with mavacamten, aficamten, and beyond

**DOI:** 10.1186/s43044-024-00587-y

**Published:** 2024-12-07

**Authors:** Khadija Sarwer, Saeeda Lashari, Nida Rafaqat, Maher, Abdul Raheem, Muneeb Ur Rehman, Syed Muhammad Iraj Abbas

**Affiliations:** 1 Liaquat University of Medical and Health Sciences, Jamshoro, Hyderabad, Sindh Pakistan; 2 Present Address: Baqai Medical University, 51, Deh Tor, Gadap Road, Near Toll Plaza, SuperHighway,, P.O. Box 2407, Karachi, 75340 Sindh Pakistan; 3 CMH Lahore Medical College & IOD, Abdur Rehman Road, Lahore Cantt, Pakistan

**Keywords:** HCM, oHCM, Mavacamten, Aficamten, Genetic mutations, Myosin inhibitors, Septal reduction therapy, Cardiac magnetic resonance imaging

## Abstract

**Background:**

A cardiac condition marked by excessive growth of heart muscle cells, hypertrophic cardiomyopathy (HCM) is a complex genetic disorder characterized by left ventricular hypertrophy, microvascular ischemia, myocardial fibrosis, and diastolic dysfunction. Obstructive hypertrophic cardiomyopathy (oHCM), a subset of HCM, involves significant obstruction in the left ventricular outflow tract (LVOT), leading to symptoms like dyspnea, fatigue, and potentially life-threatening cardiac events. With advancements in genetic understanding and the introduction of novel pharmacologic agents, including cardiac myosin inhibitors like mavacamten and aficamten, there is a paradigm shift in the therapeutic approach to oHCM.

**Main body:**

The underlying mechanisms of HCM are closely tied to genetic mutations affecting sarcomere proteins, particularly those encoded by the MYH7 and MYBPC3 genes. These mutations lead to disrupted sarcomere function, resulting in hypertrophic changes and LVOT obstruction. While genetic heterogeneity is a hallmark of HCM, clinical diagnosis relies heavily on imaging techniques such as Echocardiography and cardiac magnetic resonance imaging to assess the extent of hypertrophy and obstruction. Current pharmacological management of obstructive HCM (oHCM) focuses on alleviating symptoms rather than modifying disease progression. Beta-blockers and calcium channel blockers are primary treatment options, although their effectiveness varies among patients. Recent clinical trials have highlighted the potential of novel cardiac myosin inhibitors, including mavacamten and aficamten, in enhancing exercise capacity, reducing LVOT obstruction, and improving overall cardiac function. These innovative agents represent a significant breakthrough in targeting the fundamental pathophysiological mechanisms driving oHCM. A comprehensive literature review was conducted, utilizing top-tier databases such as PubMed, Scopus, and Google Scholar, to compile an authoritative and up-to-date overview of the current advancements in the field. This review sheds light on the updated 2024 American Heart Association (AHA) guidelines for HCM management, emphasizing the treatment cascade and tailored management for each stage of oHCM. By introducing a new paradigm for personalized medicine in oHCM, this research leverages advanced genomics, biomarkers, and imaging techniques to optimize treatment strategies.

**Conclusions:**

The introduction of cardiac myosin inhibitors heralds a new era in the management of oHCM. By directly targeting the molecular mechanisms underpinning the disease, these novel therapies offer improved symptom relief and functional outcomes. Ongoing research into the genetic basis of HCM and the development of targeted treatments holds promise for further enhancing patient care. Future studies should continue to refine these therapeutic strategies and explore their long-term benefits and potential in diverse patient populations. This review makes a significant contribution to the field by synthesizing the most recent AHA guidelines, emphasizing the crucial role of tailored management strategies in optimizing outcomes for patients with oHCM, and promoting the incorporation of cutting-edge genomics and imaging modalities to enhance personalized care.

## Background

Hypertrophic cardiomyopathy (HCM) is a complex genetic disorder stemming from dysfunctional cardiac sarcomeres, leading to excessive contractile activation and altered calcium handling [1, 2]. The hallmark characteristics of HCM comprise ventricular wall remodeling, microvascular dysfunction, scar formation within the myocardium, and abnormal diastolic function resulting from impaired ventricular compliance [1–3]. Previously thought to be rare and highly lethal, HCM now affects an estimated 1 in 500 individuals, often following an autosomal dominant inheritance pattern [1, 4–7]. Pathogenic variations occur in genes like MYH7 and MYBPC3, altering sarcomeric protein relaxation and increasing contractility and energy requirements [1–3, 7]. About 60% of patients lack detectable sarcomeric variants, with some having a family history or polygenic etiology [8, 9]. The exact molecular pathways driving ventricular hypertrophy have yet to be fully elucidated.

A considerable majority of patients (around 67%) exhibit left ventricular outflow tract (LVOT) obstruction, which substantially impacts the presentation of symptoms and drives the progression of the disease [10]. The clinical presentation of HCM is remarkably heterogeneous, encompassing a broad spectrum of symptoms and disease expressions, with patients experiencing varying degrees of symptoms like dyspnea, palpitations, and fatigue [1, 11–13].

HCM has two main forms: obstructive hypertrophic cardiomyopathy (oHCM) and non-obstructive (nHCM). oHCM is characterized by a significant obstruction in the LVOT, with a resting gradient exceeding 30 mmHg. This condition leads to various symptoms, including breathing difficulties, fatigue during physical activity, chest discomfort, and even fainting or life-threatening cardiac events. Complications include syncope, atrial fibrillation, ventricular tachycardia, stroke, heart failure, and sudden death, which can be mitigated with implantable cardioverter defibrillator placement in high-risk patients [14–18].

The diagnostic process for HCM predominantly involves clinical assessment and imaging modalities such as Echocardiography (ECHO) and cardiac magnetic resonance (CMR) imaging, which enable the detection of left ventricular hypertrophy, given the constraints of genetic testing in accurately identifying the condition [1, 19]. A left ventricular wall thickness that surpasses expected values, particularly in those with a significant familial predisposition, should raise suspicion for HCM. Given the condition's diverse morphological presentations, which often involve hypertrophy beyond the basal septum, a thorough imaging workup is essential for accurate diagnosis. A comprehensive differential diagnosis is vital in evaluating patients suspected of having HCM, as various conditions, including hypertensive heart disease, aortic stenosis, amyloidosis, muscular dystrophies, Fabry's disease, and lysosomal storage disorders, can masquerade as HCM, necessitating careful consideration to avoid misdiagnosis [1, 19].

A thorough diagnostic evaluation is crucial to differentiate HCM from these conditions and ensure accurate diagnosis and management. The detection of an intracavitary gradient is a key differentiator between oHCM and nHCM, enabling clinicians to accurately classify patients into these distinct phenotypes A resting LVOT gradient exceeding 30 mmHg is generally considered indicative of obstruction in HCM. However, in patients with a gradient less than 50 mmHg, especially those with borderline or fluctuating measurements, additional provocative testing such as exercise stress ECHO may be necessary to assess for latent obstruction, which may manifest only under exertional conditions [1, 19]. To reveal a concealed higher gradient, diagnostic provocation tests such as amyl nitrate administration, Valsalva maneuver, or exercise ECHO may be employed. These tests can help uncover the true extent of obstruction, guiding accurate diagnosis and treatment decisions [1, 19].

Obstruction in HCM is characterized by distinct features, including the abnormal systolic anterior motion of the mitral valve, which yields a posteriorly directed mitral regurgitant jet. Furthermore, CMR imaging can serve as a valuable adjunct to ECHO in equivocal cases, providing enhanced anatomical definition of hypertrophic regions and subvalvular apparatus anomalies, as well as quantifying the extent of fibrosis through late gadolinium enhancement techniques. This multimodal approach can facilitate a more comprehensive understanding of HCM pathology and improve diagnostic accuracy [1, 19].

In the management of dynamic left ventricular (LV) obstruction in HCM, pharmacological interventions prioritize symptom management and relief, as there is no compelling evidence to suggest that they influence the natural history or disease trajectory. The focus of medical therapy is to enhance patient comfort and reduce symptoms, rather than alter the fundamental course of the condition [20]. In the pharmacological management of HCM, symptom response serves as the primary indicator of treatment success, rather than changes in the measured gradient, due to the variable nature of outflow tract obstruction. The treatment algorithm begins with non-vasodilating beta-blockers, followed by calcium channel blockers (verapamil or diltiazem) as secondary options. Patients who fail to respond to these medications may be considered for advanced therapies, including disopyramide, mavacamten, aficamten, or septal reduction. Conversely, medications that exacerbate outflow tract obstruction, such as pure vasodilators and high-dose diuretics, should be avoided. Low-dose diuretics, however, may be useful in combination with other medications for patients with persistent dyspnea or congestive symptoms. Septal reduction therapy (SRT) is reserved for patients with severe, drug-resistant symptoms and should be conducted exclusively in high-volume HCM centers with demonstrated expertise. Transaortic extended septal myectomy (ESM) stands out as a premier treatment option, delivering dependable and comprehensive gradient relief at any ventricular level, accompanied by a remarkably low mortality rate (< 1%) and an outstanding clinical success rate surpassing 90–95% [20–32]. ESM eliminates or reduces systolic anterior motion-mediated mitral regurgitation and its consequences. Long-term follow-up after ESM reveals survival rates equivalent to those of the general population, with rare instances of recurrent outflow tract obstruction, supporting the efficacy and sustainability of this surgical approach. ESM is the preferred treatment option when complex cardiac conditions or papillary muscle abnormalities coexist with HCM. Alcohol septal ablation, on the other hand, offers a less invasive alternative with a low risk of procedural mortality (< 1%), but its applicability is contingent upon favorable coronary anatomy. Although it offers the benefits of reduced hospitalization and avoidance of sternotomy, its efficacy is diminished in cases with severe gradients (≥ 100 mm Hg) and thicker septal dimensions (≥ 30 mm), and it is associated with an increased risk of permanent pacemaker implantation and repeat interventions [33]. Five-year survival is similar between alcohol septal ablation and myectomy, but 10-year survival is lower with alcohol septal ablation [34–37]. Table 1 shows the comparative efficacy of pharmacological Interventions for HCM.

**Table 1:** This table compares the benefits and evidence of various drugs in treating HCM, including beta-blockers, calcium channel blockers, disopyramide, and cardiac myosin inhibitors

Treatment	Drug	Benefits	Evidence
Beta-blockers	Propranolol [47]	Symptom reduction and functional ability against a background of standard care [47]	Single-blind, placebo-controlled study [47]
	Metoprolol [48]	Reduces both resting and exercise-induced obstructed blood flow from the left heart chamber resulting in enhanced patient outcomes [48]	Insights from large patient databases and a meticulously planned, randomized, and placebo-controlled study featuring a crossover design [48]
Calcium Channel Blockers	Verapamil [49]	Upscales aerobic capacity and demonstrates similar efficacy to propranolol in comparison to placebo [49]	Randomized double-blind study [49]
	Diltiazem [50]	Enhances exercise endurance similarly to verapamil, while also alleviating symptoms [50].	Crossover trial with blinded evaluations [50]
Disopyramide [51]		Mitigates symptoms and cuts LVOT gradient in half [51]	Multi-institutional, observational research consortium [51]
Cardiac Myosin Inhibitors	Mavacamten [52]	Enhances overall cardiac well-being by boosting exercise tolerance, alleviating ventricular obstruction, and improving cardiac function in oHCM patients. Furthermore, it decreases the necessity for interventional procedures and minimizes signs of cardiac distress in nHCM patients [52]	Phases 1, 2, and 3 trials; Randomized placebo-controlled trials [52]
	Aficamten [32]	Boosts exercise capacity, reduces LVOT obstruction, and improves heart function, leading to a better NYHA classification [32].	Phase 2 trial [32]

oHCM: Obstructive Hypertrophic Cardiomyopathy, nHCM: non-obstructive Hypertrophic Cardiomyopathy, NYHA: New York Heart Association Classification

**Table 2:** Outlines the key genes implicated in HCM, detailing the associated proteins, their functions, and the genes' tolerance to missense and loss-of-function (LoF) variations, providing insights into the molecular mechanisms underlying HCM

Gene	Protein and its associated biological function	Tolerance to variation
ACTN2	Alpha actinin 2,Z disc protein	Z score: 1.76; pLI: 1.0
ANKRD1	Ankyrin repeat domain 1,Suppressor of cardiac gene expression	Z score: − 0.01; pLI: 0.00
CASQ2	Calsequestrin 2,CBP	Missense (Z score): − 1.08; LoF (pLI): 0.00
CAV3	Caveolin 3,CAP	Z score: 1.19; pLI: 0.34
JPH2	Junctophilin 2,enables the efficient transmission of calcium-mediated signals within cells, facilitating vital cellular processes	Z score: 3.93; pLI: 0.01
LDB3	LIM domain-binding 3,Z disc protein	Z score: 0.32; pLI: 0.00
MYH6	Myosin heavy chain alpha,a protein that forms part of the sarcomere, with relatively low expression levels in adult hearts, but vital for supporting cardiac muscle contraction and relaxation	Z score: 2.87; pLI: 0.00
MYLK2	Myosin light chain kinase 2,catalyzes the phosphorylation of myosin light chain 2, a crucial regulatory step in cardiac muscle contraction and relaxation	Z score: 0.73; pLI: 0.22
NEXN	Nexilin,Z disc protein	Z score: − 1.32; pLI: 0.00
TNNC1	Cardiac troponin C,a calcium-sensitive regulator that controls myofilament function, enabling precise adjustments to cardiac muscle contraction and relaxation	Missense (Z score): 2.22; LoF (pLI): 0.51
VCL	Vinculin,Z disc protein	Z score: 3.11; pLI: 0.99

LoF: Loss-Of-Function, pLi: Probabilities of Loss-of-Function Intolerance, CBP: Calcium-binding protein, CAP: Caveolae-associated protein

Historically, the risk of a sudden and lethal cardiac event has been perceived in pediatric patients with HCM has been evaluated using adult-based risk stratification tools, which may not be specifically applicable to the juvenile demographic due to differences in disease expression. However, recent research suggests that these adult-derived risk factors may not accurately predict sudden cardiac death (SCD) in pediatric patients, highlighting the need for more age-specific risk stratification approaches [34, 38–45].

Delivering comprehensive care for heart failure patients with HCM requires a unified, multidisciplinary effort, harnessing the skills of various healthcare experts. A team of specialists, including heart failure specialists, intensive care doctors, general practitioners, advanced practice providers, nurse specialists, medication management experts, patient advocates, movement and exercise specialists, and recovery and wellness experts, works together to provide seamless, coordinated support from diagnosis to treatment and recovery. In recent years (2022), treatment strategies for these patients were primarily focused on symptom management through pharmaceutical interventions or invasive procedures like septal reduction therapies, highlighting the need for innovative solutions. However, the advent of mavacamten, a pioneering drug designed to target the root causes of obstructive HCM, has introduced a novel therapeutic pathway. Mavacamten is associated with significant improvements in new york heart association (NYHA) symptomatic class and various hemodynamic and structural parameters. Its approval by the FDA [46] provides a crucial new option for treating patients with obstructive HCM and symptomatic heart failure, addressing a previously unmet need in this patient population.

A thorough insight of the pharmacodynamic and cardiovascular effects of mavacamten and aficamten is essential for personalized treatment planning, monitoring, and adjustment in patients with oHCM. Ongoing clinical investigations are expanding our knowledge of their applications in non-obstructive HCM and obstructive HCM patients who meet criteria for interventional procedures, paving the way for a deeper understanding of their therapeutic potential. This groundbreaking era of targeted HCM therapy holds tremendous promise for revolutionizing patient care and outcomes.

## Pathogenesis of OHCM: unraveling genetic and molecular mechanisms

**Fig. 1:**
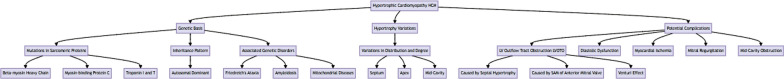
Pathogenesis, Genetic and Clinical Factors in HCM. LVO: Left Ventricular Outflow, OTO: Outflow Tract Obstruction, MR: Mitral Regurgitation, SAM: Systolic Anterior Motion, LVH: Left Ventricular Hypertrophy

HCM is a cardiac condition where the left ventricular (LV) wall exhibits excessive growth, leading to enlarged dimensions in one or more segments of the myocardium [53, 54]. The mechanism underlying LVOT obstruction in HCM involves a complex interplay of anatomical factors. Hypertrophy of the basal interventricular septum and papillary muscles reduces the cross-sectional area of the LVOT, creating a narrowed pathway for blood to exit the LV [4, 7]. Concurrently, abnormalities of the mitral valve apparatus, such as elongated or redundant leaflet tissue and anterior displacement of the coaptation line, further compromise LVOT patency [55]. As the ventricle contracts, rapid ejection through the narrowed outflow tract generates Venturi forces, causing systolic anterior motion of the mitral valve (SAM) and mitral regurgitation [10, 56]. The anterior mitral valve leaflet is drawn into the outflow tract, creating a physical barrier to blood flow and exacerbating obstruction [19, 57]. This intricate mechanism ultimately leads to increased pressure gradients across the LVOT, compromising cardiac output and increasing the risk of heart failure and SCD [56, 57]. The development of HCM cannot be explained solely by external factors like hypertension, but is instead a complex condition driven by inherent cardiac abnormalities. Genetic mutations play a significant role, with up to 60% of cases caused by mutations in sarcomeric protein-encoding genes, inherited in an autosomal dominant pattern [53]. These mutations affect critical proteins like beta-myosin heavy chain, myosin-binding protein C, troponin I and T. Furthermore, 5–10% of HCM cases are associated with other genetic disorders, including rare conditions, such as genetic storage disorders, neurodegenerative disorders, and energy production disorders [54]. The expression of HCM is defined by a wide range of hypertrophy patterns, affecting different segments of the LV, including the septal, apical, and mid-cavity regions. As the disease progresses, the excessive hypertrophy can lead to a cascade of complications, such as LVOT obstruction, diastolic dysfunction, myocardial ischemia, and mitral regurgitation, which can substantially compromise cardiac function and amplify the risk of harmful effects [53, 54]. In obstructive HCM, the classic form, septal hypertrophy and systolic anterior movement of the anterior mitral valve combine to create a Venturi effect, causing LV outflow tract obstruction [53]. Other HCM morphologic variants can also lead to mid-cavity obstruction, highlighting the complexity of this condition [54]. Figure 1 shows Genetic and Pathophysiological Factors of HCM.

The pathogenesis of HCM is a multifaceted and intricate process, involving a complex interplay of genetic, molecular, and cellular mechanisms. The diverse array of causal genes and mutations underlying HCM contributes to a heterogeneous landscape of disease manifestations, reflecting the intricate relationships between genotype, phenotype, and environmental factors. This complexity gives rise to a comprehensive collection of clinical presentations, characteristic profiles, and outcome measures, highlighting the need for personalized approaches to diagnosis, treatment, and management. These mechanisms can be categorized into four interlocking sets: primary defect, initial phenotypes, intermediate phenotypes, and tertiary effects. The primary defect is the mutation itself, triggering a cascade of downstream events. Initial phenotypes result directly from the mutation's impact on sarcomere protein structure and function [58–61]. Intermediate phenotypes represent molecular manifestations of these alterations, encompassing changes in signaling cascades, such as MAPK and TGFB1, and transcriptional reprogramming [62–72]. The downstream consequences of these molecular perturbations manifest as histological and pathological changes within the myocardium, resulting from the cascade of secondary molecular events [73–76].

The genetic mutations in HCM result in a variety of initial aberrations that disrupt sarcomere protein structure and function. These mutations instigate a series of transcriptional and translational alterations, leading to protein synthesis arrest, production of truncated polypeptides, and incorporation of aberrant proteins into the sarcomeric matrix. This triad of molecular malfunctions compromises the contractile function of cardiac muscle cells [58–61]. Aberrant protein incorporation within the sarcomeres impacts several aspects of acto-myosin function, including calcium-dependent regulation of cross-bridge dynamics. Mutations in the thin filament proteins increase Ca++ sensitivity of myofibrillar ATPase activity and force generation, further contributing to sarcomeric dysfunction [75–86]. Secondary molecular events, which include altered signaling cascades such as MAPK and TGFB1, ultimately lead to cardiac hypertrophy and characteristic morphological changes in HCM [62–72].

The tertiary phenotypes of HCM encompass the structural and histological changes that occur within the heart, including cardiac muscle fiber enlargement, cellular disorganization, and scar tissue formation [73, 74] .These changes arise as a result of the intermediary molecular events, ultimately giving rise to quaternary phenotypes, such as cardiac failure and arrhythmias.

### Molecular genetics: the underlying principles

HCM is a classic example of a genetically determined condition, typically triggered by a lone genetic mutation, which can manifest with an autosomal dominant inheritance pattern, leading to a range of disease manifestations [82]. The variability in phenotype is partly due to the interaction between the causal mutation and other genetic and non-genetic factors. Approximately 60% of HCM patients have a clear family history of the diseases [87, 88]. While autosomal recessive and X-linked modes of inheritance have been reported, they are rare [87, 88]. X-linked inheritance often raises the possibility of phenocopy conditions, such as Fabry disease [89]. Phenocopy conditions also occur in syndromic conditions like Noonan syndrome and storage diseases like Anderson-Fabry disease [89–91].

Seminal work by Christine and Jonathan Seidman laid the foundation for the understanding of HCM's molecular genetic basis. The discovery of the p.Arg403Glu mutation in the MYH7 gene, encoding β-myosin heavy chain, in a French-Canadian family [83] paved the way for subsequent important discoveries [84]. Genetic analysis has uncovered a complex pattern of mutations in key sarcomere genes, underscoring the heterogeneous nature of HCM. The genes MYH7 and MYBPC3, essential for normal cardiac function, are the most frequently implicated, together accounting for around half of all inherited HCM cases [85]. Variants in the genes TNNT2, TNNI3, and TPM1, which play vital roles in regulating cardiac muscle contraction, are infrequent causes of HCM, responsible for a small fraction (less than 10%) of all cases [86, 92–94]. Moreover, variants in the genes ACTC1, MYL2, MYL3, and CSRP3 have been implicated as infrequent, but established, contributors to HCM, highlighting the genetic heterogeneity of this disease [88–90].

HCM is caused by rare mutations, primarily affecting genes encoding sarcomere and sarcomere-associated proteins. The predominant type of mutations identified in HCM-associated genes are missense mutations, which disrupt normal protein function by substituting a single amino acid in the protein sequence, thereby altering its structure and activity [95–97]. MYBPC3 stands out due to its high propensity for mutations that lead to protein disruption, including frameshift errors, which compromise protein integrity and function [95–97]. These frameshift mutations often lead to protein degradation through the nonsense-mediated decay pathway or the ubiquitin–proteasome system, resulting in haploinsufficiency [98, 99]. Additionally, rare deletion mutations have been reported in genes such as MYH7 and TNNT2 [98, 99].

Two notable mutations, the p.Arg502Trp in MYBPC3 and the p.Val762Asp in the same gene, have been reported in relatively high frequencies within specific populations. The p.Arg502Trp mutation is found in 1.5–3% of HCM patients [100–102], while the p.Val762Asp mutation is present in 3.9% of the Japanese population [103]. The increased frequency of mutations in these regions may point to the presence of genomic hotspots or a shared genetic heritage, which could explain the higher incidence of mutations linked to HCM [101]. This observation may be cohort-specific and not necessarily generalizable to all HCM populations, as the genetic landscape of HCM can vary significantly across different populations and studies [104–106].

HCM is characterized by genetic heterogeneity, primarily due to rare mutations in genes encoding sarcomere proteins. The elucidation of these mutations has significantly advanced our understanding of the disease, with MYH7 and MYBPC3 mutations being predominant, followed by less common mutations in genes like TNNT2, TNNI3, and TPM1. The identification of these mutations has not only improved our knowledge of HCM but also highlights the complexity and genetic diversity of the disease, paving the way for personalized diagnostic and therapeutic approaches.

### Gene protein function tolerance to variation

HCM is linked to over 450 genetic mutations, mainly affecting cardiac muscle's sarcomere and myofilaments. Nevertheless, a significant proportion of HCM patients, approximately 66%, remain genetically unexplained, as only about one-third of patients test positive for a known disease-causing mutation [107]. Emerging technologies like CRISPR offer hope. A recent study successfully corrected HCM-causing mutations using endogenous DNA repair mechanisms. Gene editing may shift focus toward identifying pathogenic gene mutations and developing targeted therapies, potentially leading to a cure.

The heart's harmony relies on a symphony of genes, and research has identified 11 maestros that orchestrate cardiac muscle function: ACTN2, ANKRD1, CASQ2, CAV3, JPH2, LDB3, MYH6, MYLK2, NEXN, TNNC1, and VCL [108, 109]. These genetic virtuosos play pivotal roles in muscle structure, calcium signaling, and overall heart health. But how do they respond to genetic variations? By analyzing missense Z scores and loss-of-function probabilities (pLI), scientists can gauge their tolerance to changes.Some genes, like ACTN2 and JPH2, are perfectionists, with high intolerance to missense mutations (Z scores of 1.76 and 3.93, respectively) [109]. Even minor alterations could significantly impact their functions, like a single misplaced note in a melody. In contrast, genes like ANKRD1 and NEXN are more resilient, with negative Z scores (− 0.01 and − 1.32), suggesting they can adapt to genetic changes without severe functional consequences.The pLI values further highlight these differences. ACTN2, crucial for muscle structure, has a pLI of 1.0, indicating it is highly intolerant to loss-of-function mutations, like a missing beat in a rhythm. Conversely, MYH6 and MYLK2 have lower pLI values (0.00 and 0.22), suggesting they may tolerate such mutations better, like a flexible melody that can adapt to changes [108]. Table 2 presents a comprehensive overview of several genes, their associated proteins, functions, and their tolerance to genetic variation, as reflected by missense Z scores and loss-of-function (LoF) probabilities of loss-of function intolerance (pLI) [108, 109].

The Z score for each gene represents the deviation of observed variants from the expected number in the ExAC database. A higher positive Z score suggests that the gene is less tolerant to variation. The pLI score reflects the probability of intolerance to Loss-of-Function (LoF) variants, with a score of 1 indicating complete intolerance [108, 109].

### Circulating biomarkers in HCM: insights and implications

HCM is a complex condition characterized by polygenic and multifactorial inheritance patterns [110–112]. Recent epidemiological studies have highlighted an increase in its estimated prevalence from 1 in 500 [113] to approximately 1 in 200 individuals [114], underscoring its growing significance as a public health concern. While the overall clinical outcome is generally favorable, a distinct subgroup of patients is prone to developing severe and potentially catastrophic complications, including cardiac impairment, flow obstruction, heart rhythm disturbances, and potentially fatal cardiac incidents [115]. An in-depth analysis of the HCM Registry (HCMR) revealed significant differences in clinical and imaging profiles between patients with and without genetic sarcomere mutations [116].

**Fig. 2:**
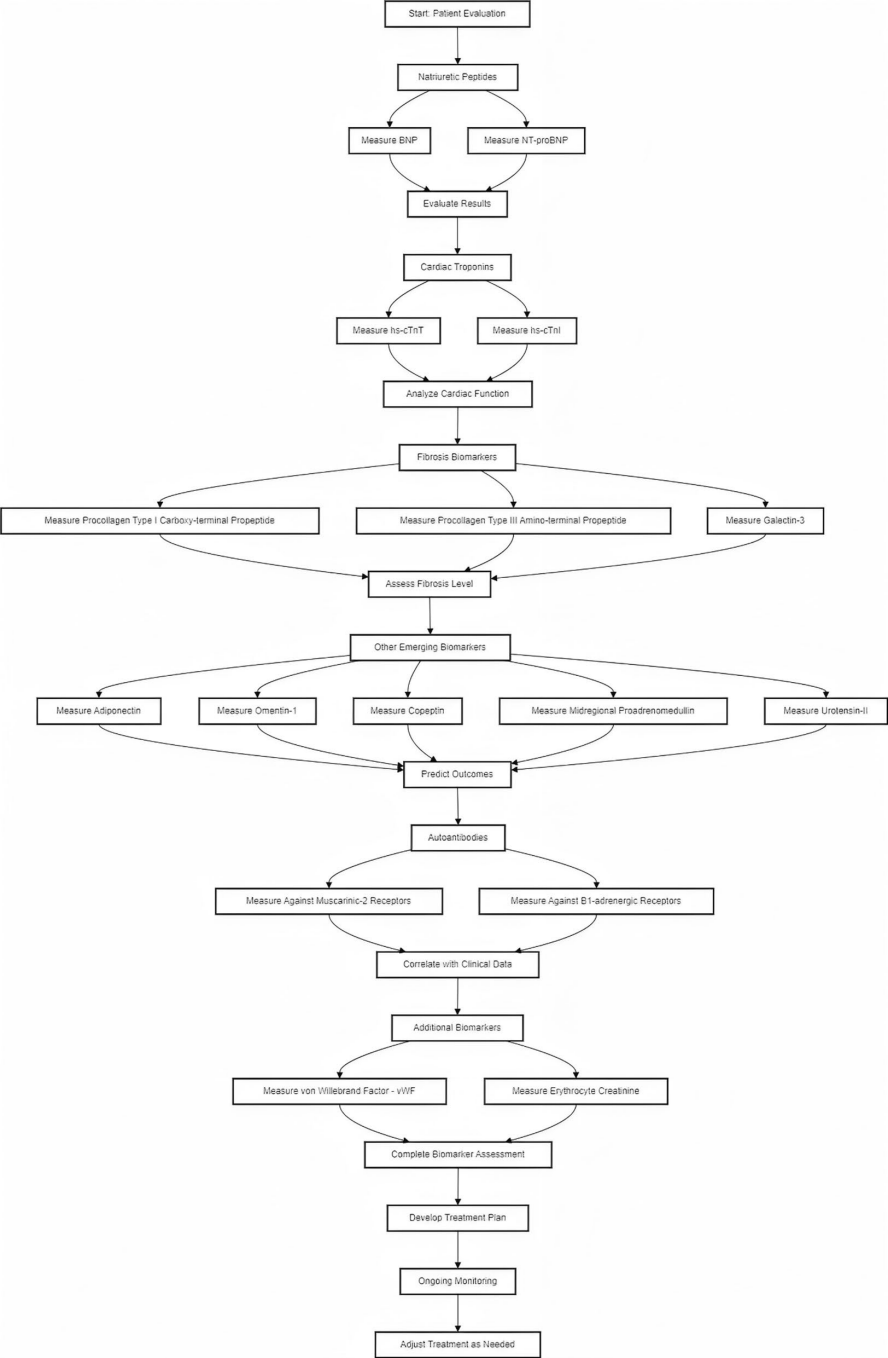
Comprehensive Biomarker Evaluation and Personalized Treatment Planning for oHCM, Cardiac Fibrosis and Function Assessment. BNP: B-type Natriuretic Peptide, NT-proBNP: N-terminal pro B-type Natriuretic Peptide, hs-cTnT: High-sensitivity Cardiac Troponin T, hs-cTnI: High-sensitivity Cardiac Troponin I, wVF: von Willebrand Factor

Regular assessment of biomarkers can facilitate early identification of disease progression, allowing for timely therapeutic interventions. Biomarkers can also guide genetic testing and family screening, as specific biomarker profiles may indicate a higher likelihood of genetic mutations. In summary, biomarkers have significantly advanced our understanding and management of HCM. Natriuretic peptides, cardiac troponins, and biomarkers of fibrosis provide critical insights into disease pathophysiology and treatment response. The potential of autoantibodies, genomic, proteomic, and metabolomic biomarkers is an exciting frontier, offering promise for future therapeutic innovations. Integrating biomarkers into clinical practice will facilitate personalized medicine, improve treatment outcomes, and enhance patient care in HCM (as shown in Fig. 2).

Elevations in natriuretic peptides, including brain-type natriuretic peptide (BNP), atrial natriuretic peptide, and N-terminal pro-B-type Natriuretic Peptide (NT-proBNP), serve as vital indicators of myocardial wall stress in HCM, enabling clinicians to gauge disease severity and monitor treatment efficacy [117]. BNP elevation is a marker of heightened cardiovascular vulnerability, predicting a higher likelihood of adverse events [118] and are predictive of NYHA functional class, the necessity for SRT [119], heart failure hospitalization [120], ventricular tachycardia [121], and mortality in patients undergoing septal myectomy [122]. NT-proBNP levels have shown correlations with LV mass, LV mass index, and late gadolinium enhancement (LGE) on CMR [123]. The HCMR data indicated that NT-proBNP levels were higher in mutation-positive patients and those with reduced LV systolic function and a resting LV outflow tract gradient ≥ 30 mmHg [124].

Cardiac troponin T and I are highly sensitive biomarkers of acute myocardial necrosis, associated with left ventricular (LV) mass, wall thickness, and fibrosis in HCM [123, 125–128]. High-sensitivity cardiac troponin T (hs-cTnT) levels serve as independent predictors of subsequent LV dysfunction and progression to end-stage HCM [129]. Elevated hs-cTnT levels also correlate with NYHA class, outflow obstruction, systolic dysfunction, and disease severity in HCM [130]. Concurrent elevations of BNP and hs-cTnT levels provides a robust predictive model for myocardial fibrosis and increased cardiovascular event risk [118, 125].

Myocardial fibrosis, a pivotal pathological process in HCM, leads to left ventricular (LV) stiffness and diastolic dysfunction. Biomarkers of fibrosis include procollagen type I carboxy-terminal, procollagen type III amino-terminal propeptide, and galectin-3 [131–137]. Galectin-3 levels are elevated in HCM patients, correlating with septal thickness, LV mass index, NYHA functional class, and sudden death risk [134–137]. Other emerging biomarkers, such as adiponectin, omentin-1, copeptin, midregional proadrenomedullin, and urotensin-II, show promise in predicting adverse outcomes, although further research is warranted [112, 115, 138–140].

The potential role of autoantibodies against self-antigens as biomarkers in HCM is gaining traction. One study reported higher concentrations of autoantibodies against muscarinic-2 and B1-adrenergic receptors in women and patients with a history of syncope, correlating with resting LV outflow tract gradient, maximal wall thickness, and interventricular septum thickness [95, 141]. Advancements in genomic, proteomic, and metabolomic biomarkers are ongoing, with studies demonstrating the up- or downregulation of microRNAs associated with LV hypertrophy, fibrosis, and cardiomyocyte apoptosis [142]. However, their ability to predict clinical outcomes remains to be fully established.

Biomarkers linked to LV outflow tract obstruction in HCM include vWF(von Willebrand factor), hs-cTnT, NT-proBNP, erythrocyte creatinine, and copeptin [124, 130, 140, 143–145]. These biomarkers are not only indicative of LV outflow tract obstruction but also predictive of adverse clinical outcomes, such as worsening NYHA class and atrial and ventricular arrhythmias. Elevated vWF levels have been shown to accurately discriminate between patients with obstructive and non-obstructive HCM and normalize after successful surgical myomectomy [143].

Furthermore, the EXPLORER-HCM trial demonstrated that high-sensitivity hs-cTnI and NT-proBNP levels significantly decreased in response to medical therapy [146]. The VANISH trial found that patients treated with valsartan exhibited improved cardiac structure and function, along with stable or reduced NT-proBNP levels [99]. Similarly, the VALOR-HCM trial indicated that mavacamten treatment resulted in reduced NT-proBNP and cTnI levels, alongside improved NYHA functional class [147].

Biomarkers play a vital role in monitoring disease progression in HCM, allowing for the early detection of changes in disease status, assessment of treatment response, and optimization of patient care through timely interventions and adjustments to management strategies. For instance, serial measurements of NT-proBNP and hs-cTnI can detect worsening cardiac function and predict adverse outcomes [148]. Additionally, galectin-3 levels may increase with advancing disease stages [137]. Regular assessment of these biomarkers could facilitate early identification of disease progression, allowing for timely therapeutic interventions.

Considering the heterogeneity of HCM, the identification of biomarkers is crucial for personalized medicine, enabling the tailoring of treatment strategies to individual patients and potentially leading to more effective and targeted management of the disease**. **For example, patients with elevated BNP levels might benefit from more aggressive medical therapy or earlier consideration of SRT [119]. Conversely, those with normal BNP levels may require less intensive monitoring and treatment. Biomarkers can also guide genetic testing and family screening, as specific biomarker profiles may indicate a higher likelihood of genetic mutations [126].

### HCM management: a tailored strategy for each stage

Genetic testing has identified individuals with HCM-causing mutations who do not yet exhibit symptoms. This presents an opportunity to explore preventative measures. While gene editing technologies like CRISPR/Cas9 have shown promise in correcting HCM-causing mutations in human embryos, pharmacological approaches are more likely to enter clinical practice. For example, losartan, a TGF-β inhibitor, has prevented hypertrophy development in animal models [149, 150] and the VANISH trial demonstrated that valsartan can improve cardiac structure and function in individuals with early HCM phenotype, making Stage I: Pre-Phenotype Expression and Prevention a crucial period for intervention.

The quintessential HCM phenotype involves LVOTO (left ventricular outflow tract obstruction) caused by systolic anterior motion of the mitral valve. To manage LVOTO and associated symptoms, drugs with negative inotropic effects, such as beta-blockers (e.g., nadolol, metoprolol) [151, 152], non-dihydropyridine calcium channel blockers, and disopyramide, are commonly used, and are the mainstay of treatment for Stage II: Characteristic HCM with No Progression.

A significant proportion of patients (up to 15%) may exhibit the development of structural heart changes, including left ventricular fibrosis, which can compromise cardiac performance and lead to decreased diastolic and systolic function.. Attempts to target replacement fibrosis with anti-fibrotic drugs like spironolactone and losartan have been unsuccessful [149, 150] Ranolazine, an anti-anginal medication, may mitigate microvascular ischemia-driven fibrosis [153, 154] Cardiac resynchronization therapy has been considered in patients with systolic dysfunction, though data is limited, highlighting the challenges of managing Stage III: Adverse Remodeling.

A small proportion of HCM patients (5–8%) may experience significant left ventricular systolic impairment (EF < 50%), which carries a poor prognosis. In these cases, a comprehensive medical therapy approach, including angiotensin receptor-neprilysin inhibitors, beta-blockers, and SGLT2 inhibitors, should be considered to mitigate disease progression [1, 155]. For patients with refractory disease, more invasive options like LVAD (Left Ventricular Assist Device) implantation or cardiac transplantation may be required to provide adequate circulatory support and improve outcomes [156]. Across all stages, management of atrial fibrillation, a common complication, is crucial, with rhythm control preferred over rate control [1, 157] emphasizing the importance of Stage IV: Overt Systolic Dysfunction.

**Fig. 3:**
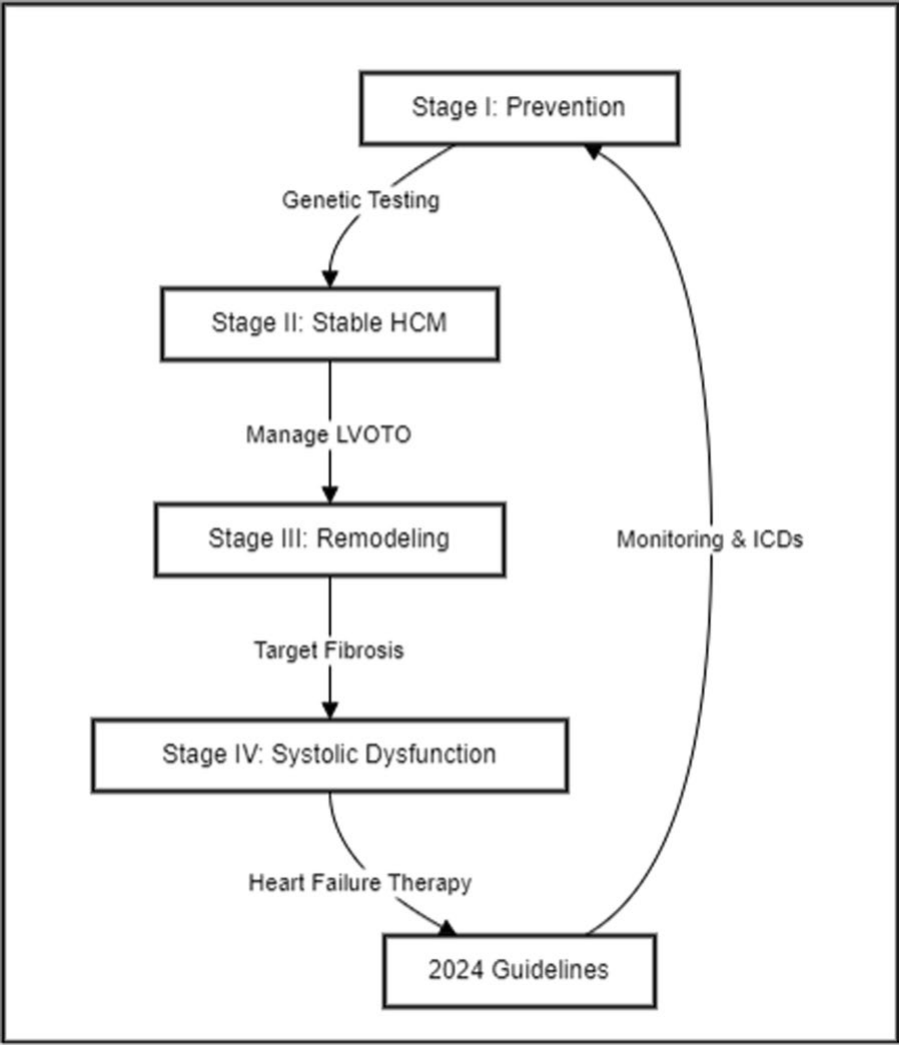
HCM Management Cascade According to 2024 Guidelines. HCM: Hypertrophic Cardiomyopathy; LVOTO: Left Ventricular Outflow Tract Obstruction; ICDs: Implantable Cardioverter-Defibrillators

According to the 2024 Guidelines [20] genetic testing is suggested for individuals with a family history of HCM or those exhibiting symptoms suggestive of HCM, to uncover potential genetic mutations that may be driving the condition [20]. ECHO is the primary mean of diagnosing and tracking HCM, offering essential information for patient care and management. CMR imaging is recommended for further evaluation of HCM in select cases. Exercise stress testing is recommended to assess functional capacity and detect potential cardiac complications. Implantable cardioverter-defibrillators (ICDs) are recommended for primary prevention of SCD in high-risk patients. Cardiac resynchronization therapy (CRT) is recommended for patients with systolic dysfunction and conduction disturbances [20]. Figure 3 shows a tailored strategy for each stage in HCM management.

### Overview of Mavacamten's global therapeutic impact

Mavacamten's global reach, spanning regulatory approvals in North America, Europe, and parts of Asia, has yielded promising real-world outcomes. Its safety and efficacy have been consistently demonstrated across trials and post-approval use, benefiting diverse patient demographics. This increased availability alleviates the therapeutic gap for undiagnosed or untreated patients, particularly in regions historically lacking access to cutting-edge cardiovascular therapies [158].

Mavacamten represents a paradigm-shifting treatment modality specifically tailored for patients experiencing debilitating symptoms of NYHA class II or III obstructive HCM. The approval of mavacamten is firmly grounded in the compelling evidence generated by the EXPLORER-HCM trial, a seminal investigation that unequivocally demonstrated the medication's superior clinical efficacy and safety profile in this distinct patient cohort. This comprehensive and randomized study showcased mavacamten's exceptional efficacy in alleviating symptoms, enhancing physical performance, and reducing the reliance on invasive procedures among patients with oHCM [159].

The translation of mavacamten from clinical trials to real-world practice has demonstrated substantial therapeutic benefit, characterized by improved clinical outcomes and a stable safety profile across varied patient demographics. This increased accessibility has bridged a critical gap in cardiovascular disease management, providing relief to a substantial number of undiagnosed or untreated patients worldwide, especially in areas with limited access to specialized therapies [158].

### Mechanism of action of Mavacantem

**Fig. 4:**
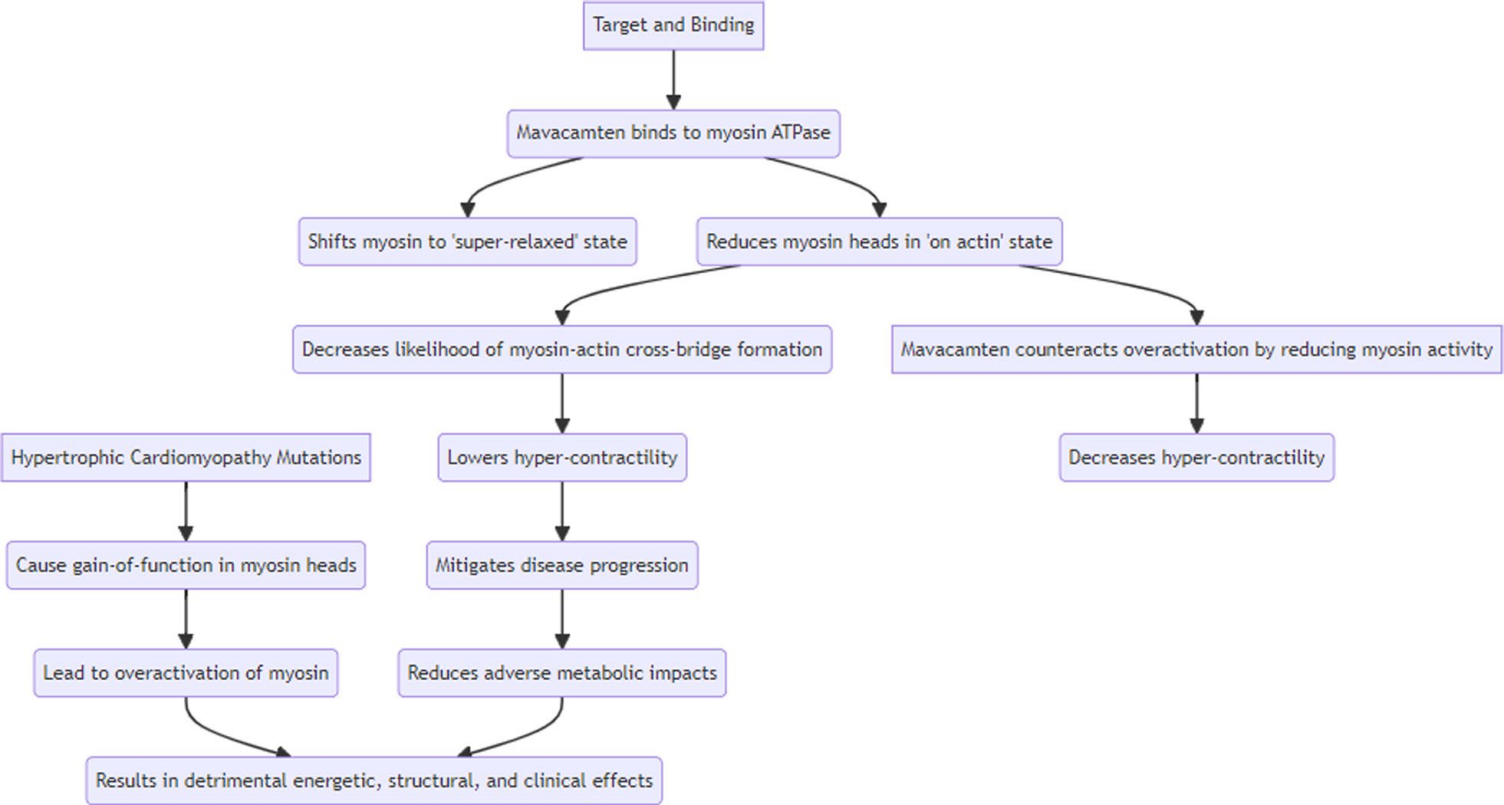
Mechanism of Action of Mavacamten in HCM. HCM: Hypertrophic Cardiomyopathy; ATPase: Adenosine Triphosphatase

Preclinical studies have unveiled mavacamten's unique mechanism of action, which involves modulating the myosin-actin interaction to reduce hypercontractility and its associated detrimental effects [160–163]. This myosin ATPase inhibitor induces a conformational shift in the myosin population, favoring a relaxed, energy-conserving state dissociated from actin, which in turn decreases cross-bridge formation and slows disease progression and symptom severity (as shown in Fig. 4) [161–164]. A cutting-edge approach counterbalances the deleterious effects of HCM-related mutations, which lead to overactive myosin heads and resultant energetic, structural, and clinical dysfunction. By harmonizing myosin head activity, this method reduces sarcomeric hyperexcitability and myocardial overcontraction, culminating in a profound improvement in cardiac function and overall cardiac well-being [161, 164–167]. By reducing LVOT obstruction, left ventricular filling pressure, maximal force, Ca2 + sensitivity, myocardial energy demands, and diastolic dysfunction, mavacamten showcases its potential as a comprehensive treatment for oHCM. Research in a feline model of oHCM has demonstrated mavacamten's efficacy in inhibiting myosin ATPase and reducing outflow tract obstruction, paving the way for further investigation into its disease-modifying effects on the structural abnormalities characteristic of oHCM. Mavacamten's pharmacokinetic profile is marked by high oral bioavailability (85%), unaffected by food intake, and extensive binding to plasma proteins (97%) [163, 168]. Metabolism is primarily mediated by CYP2C19, with secondary contribution from CYP3A4, and elimination occurs predominantly through renal excretion (over 80%), with the remaining fraction excreted via the gastrointestinal tract [168].

### Exploring mavacamten: an in-depth analysis of clinical trials

Notably, the EXPLORER-HCM trial validated mavacamten's effectiveness in enhancing cardiopulmonary function and alleviating symptoms in oHCM patients. These findings highlight mavacamten's potential to significantly improve therapeutic outcomes in this challenging condition. The pharmacokinetic profile of mavacamten was thoroughly assessed in Phase 1 trials, which revealed rapid absorption and extensive metabolism via cytochrome P450 enzymes, particularly CYP2C19 and CYP3A4.The terminal half-life of the drug varies with CYP2C19 metabolic status, ranging from 6 to 23 days. Additionally, the use of CYP2C19 and CYP3A4 inducers or inhibitors can impact mavacamten's systemic exposure, necessitating careful consideration when co-administering medications that affect these enzymes [168, 169].

The PIONEER-HCM trial [170], a 12-week Phase 2 study examined the safety and efficacy of an investigational treatment in 21 patients with symptomatic oHCM. The open-label trial featured two separate cohorts: Cohort A started with a daily dose of 10 or 15 mg, with possible dose adjustments at week 4 to achieve a predetermined 15–20% reduction in resting LVEF from baseline, while Cohort B initiated treatment at a lower dose (2 mg/day) and could increase to 5 mg/day if the resting LVOT gradient did not decrease by more than 50% from baseline [170].

Results from the PIONEER-HCM trial indicated significant reductions in post-exercise LVOT gradients at Week 12, with Cohort A showing a mean change of − 89.5 mmHg and Cohort B -25.0 mmHg [170]. Further analysis revealed significant enhancements in secondary endpoints, including pressure gradients at rest and during Valsalva maneuvers, aerobic capacity (peak oxygen consumption), ventilatory efficiency (VE/VCO2 slope), and symptom severity (dyspnea scores). A therapeutic window of plasma concentrations (350–695 ng/mL) was identified, where substantial reductions in LVOT obstruction occurred without compromising left ventricular ejection fraction (> 50%). However, higher plasma levels (> 695 ng/mL) led to more marked declines in left ventricular ejection fraction, ranging from 34 to 49% [170]. Most reported adverse events were transient and did not appear to be related to the study medication, indicating that the treatment was well-tolerated by participants.

Participants from the PIONEER-HCM trial were subsequently invited to join the PIONEER-OLE open-label extension study [171], where mavacamten was initiated at 5 mg/day post-washout. Dosages were adjusted to achieve a plasma concentration of 250–500 ng/mL, resulting in sustained improvements in LVOT obstruction, NYHA functional class, and NT-proBNP levels at 48 weeks [171]. Long-term follow-up at 3 years demonstrated continued improvements in cardiovascular hemodynamics, symptoms, and quality of life. Additionally, AI-enhanced electrocardiography (AI-ECG) analysis showed decreasing mean HCM scores over time, reflecting improvements in ECG morphology. These improvements positively correlated with reductions in Valsalva LVOT gradients and NT-proBNP levels, indicating favorable disease status measures [172].

Building on the encouraging results from the PIONEER-HCM study, mavacamten advanced to the crucial Phase 3 EXPLORER-HCM study, a comprehensive global investigation involving 251 individuals with oHCM, characterized by preserved left ventricular ejection fraction (> 55%) and significant functional impairment, across 68 sites in 13 countries [52, 165]. Participants, mostly on beta-blocker or calcium channel blocker therapy, were initiated on mavacamten at 5 mg/day, with dose adjustments at Weeks 8 and 14 to achieve targeted therapeutic endpoints [165]. The primary endpoint at Week 30 was a composite measure of exercise capacity and symptom burden, focusing on peak oxygen consumption and NYHA class improvements [52]. The results demonstrated significant efficacy of mavacamten compared to placebo, with marked improvements in LVOT gradients, NYHA class, and patient-reported outcomes such as KCCQ-CSS and HCMSQ-SoB scores [52]. Mavacamten showed a positive effect on biomarkers of cardiac stress and injury, with NT-proBNP and hs-cTnI levels suggesting a decrease in cardiac strain [52]. Beyond these established benefits, the innovation of mavacamten lies in its Proprietary functional mechanism. As a selective cardiac myosin inhibitor, mavacamten directly targets the underlying pathophysiology of oHCM by reducing myocardial contractility and thereby decreasing LVOT obstruction. This targeted approach contrasts with traditional therapies that primarily address symptoms rather than the root cause of the disease. The integration of advanced pharmacokinetic monitoring and AI-enhanced diagnostic tools in the ongoing management of patients underscores mavacamten's role in personalized medicine. Table 3 This detailed examination aggregates and scrutinizes the findings from clinical trials targeting HCM, facilitating a comparative evaluation of treatment benefits and risks to guide therapeutic decision-making.

**Table 3:** Comparative Summary of Clinical Studies on HCM

Study (reference)	PIONEER HCM [170, 171]	EXPLORER HCM [165, 166]	VALOR-ACH [147]
Study configuration	Open-label, non-randomized	Double-blind, randomized	Double-blind, randomized
Participant count	21	251	112
Study length (weeks)	12	30	16
Heart failure severity	II/III	II/III	III/IV
Daily dose (mg)	2–20	2.5–15	2.5–15
Main study objective	Exercise-induced LVOT gradient improvement	Enhanced physical performance and reduced symptom severity	Assessing persistent qualification for SRT
Major findings	LVOT obstruction shows a significant decrease	Reduced LVOT obstruction	Decreased reliance on SRT
	Exercise capacity and breathing efficiency improve substantially	Increased exercise tolerance	Improved LVOT obstruction with reduced gradients
	Heart failure symptoms alleviate, leading to a lower NYHA classification	Improved heart failure classification (NYHA)	Enhanced heart failure symptoms with lower NYHA classification
	Breathlessness severity decreases, as indicated by lower NRS scores	Lower levels of biomarkers (NT-proBNP and hs-cTnI) indicating reduced cardiac stress	Significant reduction in biomarkers (NT-proBNP and hs-cTnI) indicating improved cardiac health
	Overall health and well-being exhibit notable enhancement	Improved heart relaxation and filling (diastolic function)	Overall improvement in health and well-being

Safety analysis revealed manageable adverse events, with temporary drug discontinuation occurring in a few cases due to LVEF considerations, though most patients completed the trial [52]. Subgroup analyses suggested consistent benefits across various patient characteristics, emphasizing mavacamten's potential as a viable treatment option for oHCM [165, 173]. Mavacamten's robust performance in EXPLORER-HCM supports its role in improving cardiopulmonary function and symptom management in oHCM patients, offering promise for enhanced therapeutic outcomes in this challenging condition.

### Dosage and administration

Oral administration of mavacamten is achieved through capsules offered in various dosages, including 2.5 mg, 5 mg, 10 mg, and 15 mg. The suggested starting point for treatment is a single daily dose of 5 mg. [170]. Dose adjustments may occur at weeks 4, 8, and 12 following treatment initiation, with a maximum approved daily dose of 15 mg. The target plasma concentration ranges from 350 ng/mL to 700 ng/mL, and achieving steady-state levels and therapeutic effects may take several weeks [170]. Regular assessment of the patient's clinical status, left ventricular ejection fraction (LVEF), and LVOT gradient is essential before dose titration [170]. Table 4 shows the mavacamten dosing regimen [170].

**Table 4:** Dosage (mg) and administration recommendations for mavacamten, including starting dose, titration options, and maximum daily dose

Dosage (mg)	Administration	Recommended Use
2.5	Oral capsule	–
5	Oral capsule	Starting dose, may titrate
10	Oral capsule	Titration possible
15	Oral capsule	Maximum daily dose

Mavacamten management strategies and criteria, as observed in the EXPLORER-HCM and VALOR-HCM trials, involve a uniform starting dose of 5 mg, with subsequent adjustments based on specific clinical indicators. Dose reduction is considered if mavacamten plasma concentrations fall within a certain range (700–1,000 ng/mL) in EXPLORER-HCM, while VALOR-HCM adds an additional criterion of a Valsalva LVOT gradient below 30 mm Hg by week 4. Upward titration is guided by achieving a resting LVEF of 50% or higher, mavacamten plasma levels below 350 ng/mL, and a Valsalva LVOT gradient of 30 mm Hg or higher at specified intervals. Temporary discontinuation is warranted if LVEF drops below 50%, mavacamten plasma concentrations exceed 1000 ng/mL, or QTcF prolongation occurs. These criteria highlight the importance of close monitoring and personalized treatment adjustments to optimize mavacamten therapy in patients with HCM [147, 174]. Table 5 shows the comparison of dosing criteria for mavacamten in EXPLORER-HCM and VALOR-HCM clinical trials, including starting dose, down-titration, up-titration, and temporary discontinuation criteria [147, 174].

**Table 5:** Mavacamten Dosing Criteria in HCM Clinical Trials

Characteristic	EXPLORER-HCM (Day 1 to Week 30)	VALOR-HCM (Day 1 to Week 16)
Initial dose of mavacamten	5 mg	5 mg
Criteria for dose reduction	Mavacamten plasma levels between 700 and 1000 ng/mL at any visit	Mavacamten plasma levels between 700 and 1000 ng/mL, or Valsalva LVOT gradient under 30 mm Hg at Week 4
Criteria for dose increase	Resting LVEF at least 50%, Mavacamten plasma levels under 350 ng/mL, and Valsalva LVOT gradient at least 30 mm Hg at Weeks 8 and 14	Resting LVEF at least 50%, Mavacamten plasma levels under 350 ng/mL, and Valsalva LVOT gradient at least 30 mm Hg at Weeks 8 and 12
Temporary stoppage criteria	Resting LVEF below 50%, Mavacamten plasma levels at or above 1000 ng/mL, or QTcF prolongation	Resting LVEF below 50%

HCM: Hypertrophic Cardiomyopathy, LVEF: Left Ventricular Ejection Fraction, LVOT: Left Ventricular Outflow Tract, QTcF: QT interval corrected for heart rate using Fridericia's formula

### Adverse effects

Key safety considerations include dizziness (27%) and syncope (6%), which were commonly reported adverse events in clinical trials [165, 170]. Additionally, mavacamten may cause a decrease in left ventricular ejection fraction (LVEF), with up to 10% reduction reported. This decrease can lead to temporary treatment discontinuation, as seen in 3.6% of patients in the EXPLORER-HCM trial and 3.6% in the VALOR-HCM trial. Other Mavacamten may cause various adverse effects, including cardiovascular issues (stress cardiomyopathy, arrhythmias, chest pain), respiratory symptoms (shortness of breath), and general complaints (headache, fatigue, leg swelling). Regular surveillance of patients is vital to identify and manage potential side effects, enabling timely adjustments to treatment plans and optimizing patient outcomes [165, 170]. Pharmacists play a crucial role in reviewing patient regimens for potential drug interactions, and patients are required to undergo regular echocardiograms during therapy [46, 52]. Although mavacamten is frequently administered alongside beta-blockers or non-dihydropyridine calcium channel blockers, it is not considered a frontline therapy for oHCM, particularly in light of recent studies that underscore the benefits of beta-blockers [151].

### Incorporating Mavacamten into treatment plans for NYHA class II/III HCM

**Fig. 5:**
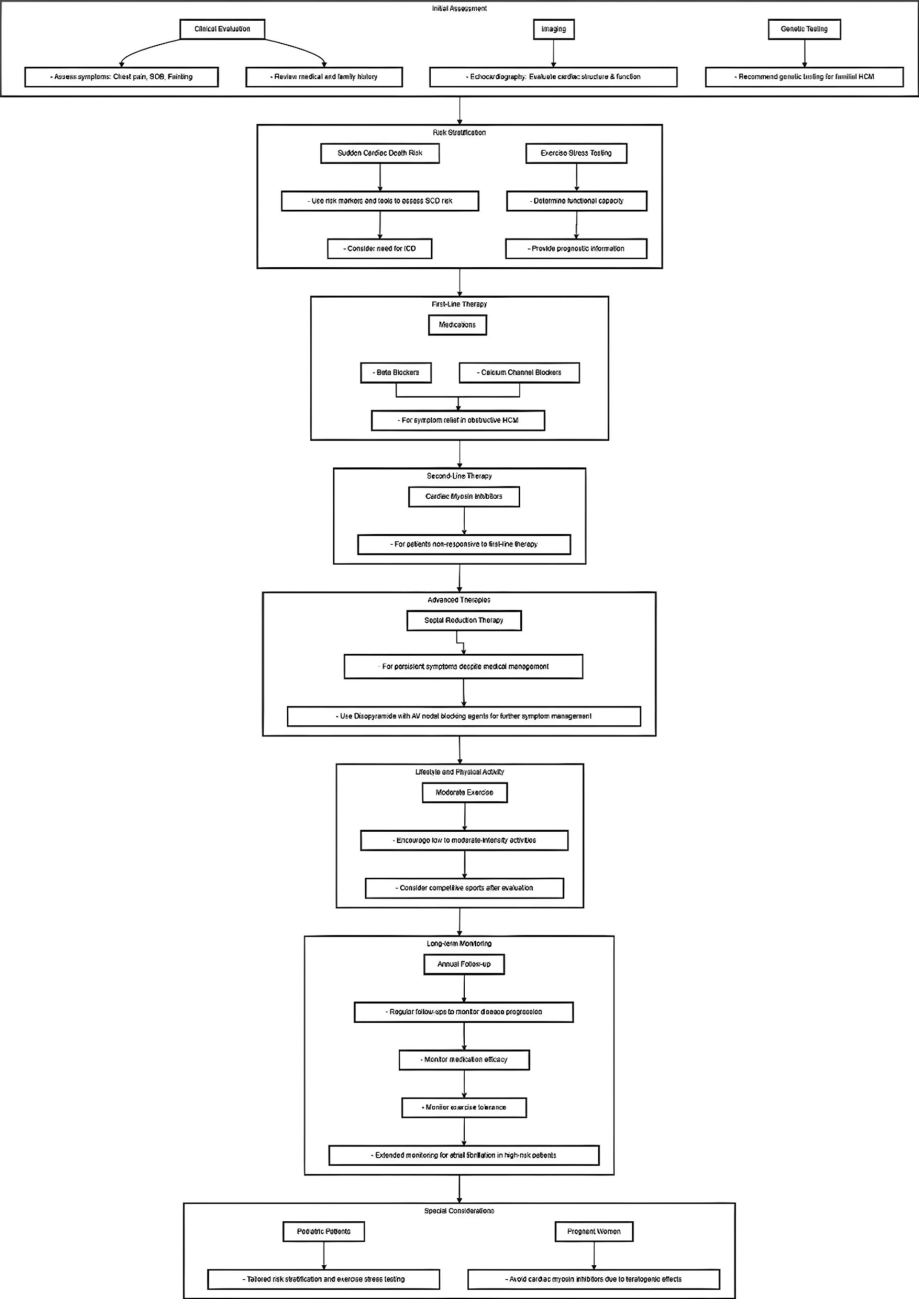
Management Algorithm for HCM. SCD: Sudden Cardiac Death, ICD: Implantable Cardioverter Defibrillator, HCM: Hypertrophic Cardiomyopathy, AV: Atrioventricular

Contrary to previous guidelines [1], the latest treatment cascade for HCM from the American Heart Association (AHA) and American College of Cardiology (ACC) now includes cardiac myosin inhibitors (CMIs) as a treatment option [20]. As shown in Fig. 5, this updated approach reflects the latest recommendations, which differ from the 2023 study [175]. CMIs are now incorporated into the treatment algorithm, offering a new option for managing HCM. Mavacamten treatment has been linked to enhanced NYHA symptom classification and notable improvements in various hemodynamic and structural parameters. The FDA's approval of mavacamten for use in patients with oHCM and symptomatic heart failure offers a novel treatment option for this specific heart failure population, providing a fresh therapeutic approach to manage their condition. Mavacamten's capacity to substantially diminish the symptom burden, thus providing a valuable therapeutic window for patients who might otherwise face the prospect of surgery.While clinical trials have highlighted short-term side effects, the long-term effects, including impacts on lactation, pregnancy, and left ventricular ejection fraction (LVEF), warrant further investigation [175, 176]. The intensive monitoring required for mavacamten and its projected economic costs pose practical challenges [46, 175]. Furthermore, certain patient groups, including those with atrial fibrillation or advanced liver or kidney disease, were excluded from major trials, leaving uncertainties about mavacamten's safety in these populations [147, 165, 170]. As such, future research must address these gaps, particularly in diverse patient populations with coexisting conditions.

**Fig. 6:**
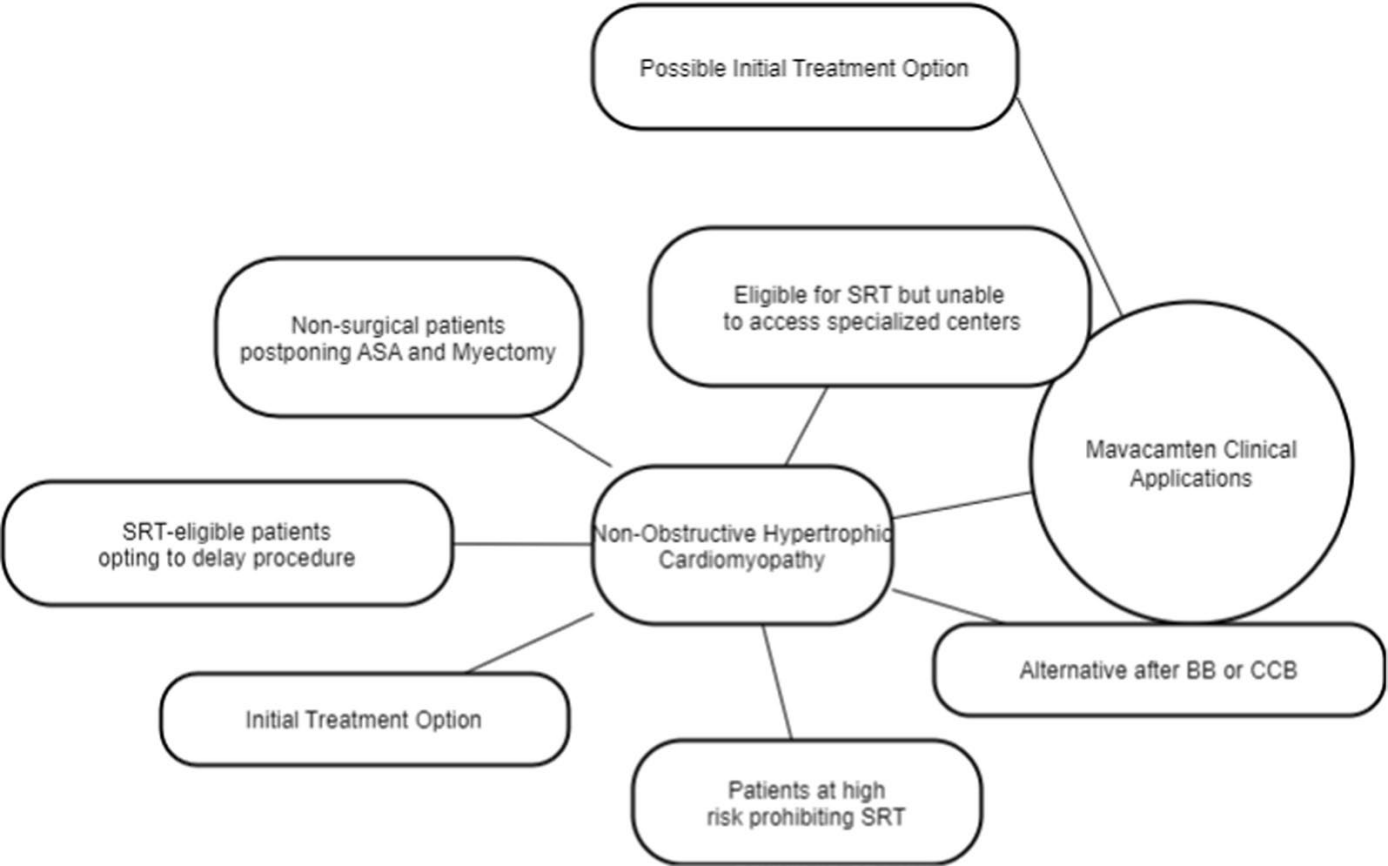
Mavacamten used in non-oHCM cases under different clinical scenarios. ASA: Alcohol Septal Ablation, SRT: Septal Reduction Therapy, BB: Beta-Blockers, CCB: Calcium Channel Blockers

Mavacamten's integration into clinical practice will be contingent upon various clinical considerations, including the effectiveness of established medical therapy, patient suitability for SRT, and tolerance to mavacamten. The FDA's approval of mavacamten for treating oHCM with progressive symptoms [46] is poised to significantly impact the treatment algorithm for oHCM patients. Currently, mavacamten is only available through the Risk Evaluation and Mitigation Strategy (REMS) program, ensuring careful monitoring and safe administration of the medication. Figure 6 shows clinical applications of mavacamten.

### Aficamten for OHCM

Aficamten, a cutting-edge myosin inhibitor, is being investigated as a potential breakthrough treatment for HCM, a condition characterized by abnormal thickening of the heart muscle. By targeting the underlying pathophysiology of HCM, aficamten aims to provide a novel and effective therapeutic strategy for patients with this disease [177]. Aficamten's pharmacokinetic properties are noteworthy, featuring a shorter half-life than mavacamten, enabling faster titration to optimal doses. Additionally, aficamten achieves steady-state concentrations within a relatively short period of two weeks, and its wide therapeutic window suggests a favorable balance between efficacy and safety, providing clinicians with flexibility in dosing and minimizing the risk of adverse reactions [177]. In the phase II REDWOOD-HCM trial, high-dose aficamten demonstrated a favorable safety profile and led to a remarkable 93% response rate, defined as a final resting LVOT gradient ≤ 30 mmHg and Valsalva LVOT gradient ≤ 50 mmHg, compared to 8% in the placebo arm [178].

Aficamten has been shown to reduce myosin ATPase activity and contractility in preclinical studies [177]. Although its effects on the super-relaxed (SRX) state of myosin are unknown, aficamten binds to the ATP-binding pocket, similar to blebbistatin, suggesting a distinct myosin off-state [177]. Preliminary data indicate that aficamten reduces cardiac contractility in vivo, similar to mavacamten, in healthy rats, beagle dogs, and a transgenic mouse model of HCM [179–181]. Pharmacokinetic assessments reveal faster kinetics than mavacamten, with a predicted human half-life of 2.8 days and actual half-life of 3.4 days, and no significant effect on cytochrome P450 [177, 182].

Preliminary results from the REDWOOD-HCM trial demonstrate thataficamten treatment leads to a substantial reduction in resting LVOT gradients and plasma NT-proBNP levels, demonstrating superiority over placebo. Notably, aficamten is well-tolerated, with a benign side effect profile and no reported serious adverse events, indicating a positive benefit-risk ratio and potential as a valuable therapeutic agent [183]. A phase 2 open-label trial evaluated aficamten's safety and efficacy in non-HCM patients. Symptomatic patients received aficamten for 10 weeks, with doses adjusted based on left ventricular ejection fraction (LVEF). Results showed 55% of patients experienced significant symptom improvement, with 29% becoming asymptomatic. Quality of life and cardiac biomarkers (NT-proBNP and troponin I) also improved. Modest LVEF reductions occurred, with three patients experiencing asymptomatic decreases below 50% (resolved after washout). One patient with a history of SCD experienced a fatal arrhythmia [178] (see discussion below). Aficamten therapy for symptomatic nHCM demonstrated a favorable safety profile and was linked to significant improvements in heart failure symptoms and cardiac biomarkers [178].

## Discussion

The recently updated guidelines for HCM diagnosis and treatment reflect a significant shift in clinical practice, incorporating novel research insights and innovative care approaches to enhance patient outcomes. Recent revisions have integrated contemporary strategies for management and treatment, poised to transform the field and yield improved patient outcomes. This update heralds a new era in the clinical approach to this complex condition, and its implications warrant thorough examination and consideration in future research and practice [20].

The standard of care for oHCM has remained largely unchanged for decades [1] with recent update adding more contemporary strategies for management and treatment [20], relying on beta-blockers [184] and/or non-dihydropyridine calcium channel blockers to alleviate symptoms by slowing heart rate and mildly decreasing contractility [1]. Disopyramide, an antiarrhythmic, may be added to enhance negative inotropic effects, but its use is often limited by anticholinergic side effects. Despite their widespread use, these medications have limited efficacy, supported mainly by observational studies, and fail to decipher the molecular pathways underlying oHCM to develop effective interventions driving the disease. SRT, including septal myectomy [185] or alcohol septal ablation [186], is restricted to treatment-refractory patients, but its use is hampered by operator expertise requirements and may not suit patients with comorbidities or preferences for non-invasive options. This significant unmet need for effective and safe medical management of oHCM has sparked a pressing demand for innovative solutions.

A phase 2 trial (NCT01912534) demonstrated valsartan's efficacy in early-stage sarcomeric HCM, improving left ventricular wall thickness and diastolic function (composite z-score + 0.231; *P* = 0.001). This complements recent studies on novel cardiac myosin inhibitors, mavacamten and aficamten, showing improved symptoms and reduced LVOT obstruction in obstructive HCM [187] .Furthermore, the TEMPO study, a double-blind, placebo-controlled, randomized trial, highlighted metoprolol's efficacy in oHCM. Metoprolol significantly reduced LVOT gradients at rest and during exercise, and improved symptom relief, with fewer patients in higher NYHA and CCS classes, and improved quality of life scores (KCCQ-OSS). However, metoprolol did not significantly affect maximum exercise capacity, underscoring its limitations in certain clinical scenarios [151].

As of the 2024 guidelines of management oHCM, cardiac myosin inhibitors are an integral part in the management of the disease [20]. Mavacamten, a pioneering selective cardiac myosin inhibitor, revolutionizes the treatment landscape by targeting the core pathophysiological mechanism of oHCM [1], offering a disease-specific approach that tackles the root cause of the disease.

By targeting the underlying pathophysiology, mavacamten offers a crucial alternative for patients refractory to conventional therapies. Emerging evidence highlights its efficacy in symptom alleviation and potential to delay or prevent SRT. However, its use requires careful management due to the risk of LV systolic dysfunction, necessitating a Risk Evaluation and Mitigation Strategy (REMS) program. This innovative approach ensures provider certification, patient education, and comprehensive oversight, Creating a framework for the development of a new treatment algorithm in oHCM [46, 52].

**Fig. 7:**
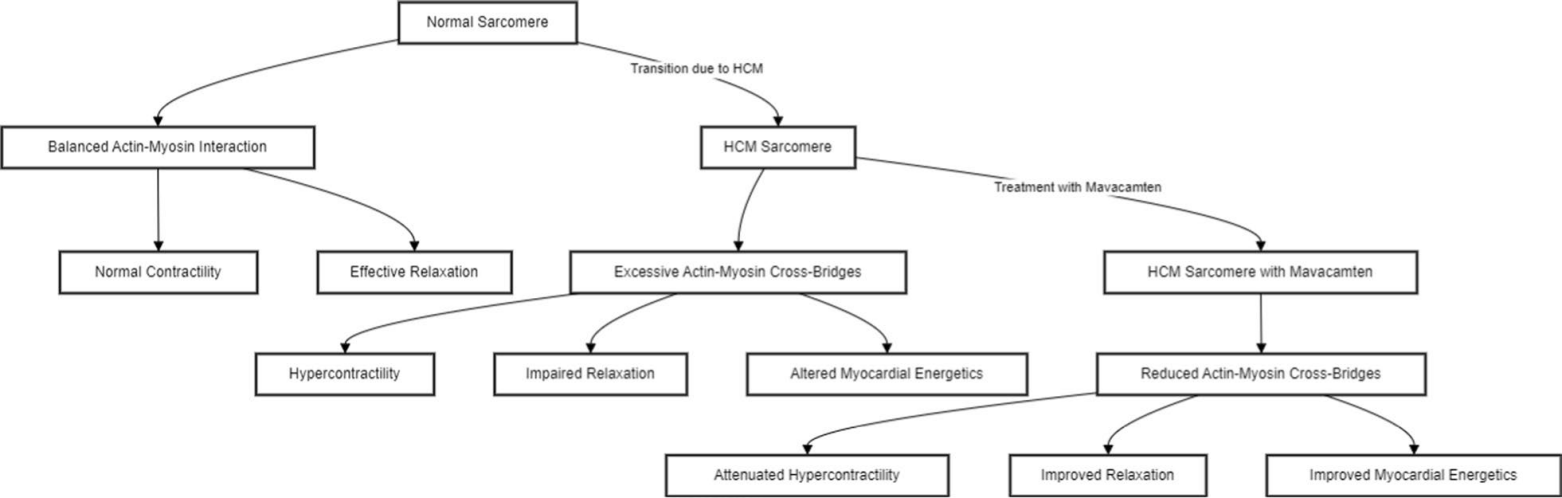
Shows the transition from a normal sarcomere to an HCM sarcomere and the effects of mavacamten treatment on contractility, relaxation, and myocardial energetics

Figure 7 illustrates the mechanism of action of myosin inhibitors like mavacamten across three distinct states of sarcomere function: Normal, HCM, and HCM with mavacamten treatment. In a healthy sarcomere, the interaction between actin and myosin filaments is well-balanced, facilitating optimal muscle contractility and relaxation, thereby ensuring normal cardiac function. Conversely, in HCM sarcomeres, an excessive formation of actin-myosin cross-bridges results in hypercontractility and impaired relaxation, disrupting normal cardiac function and altering myocardial energetics. Mavacamten's intervention in HCM sarcomeres reduces the number of actin-myosin cross-bridges, mitigating hypercontractility and enhancing relaxation. This restoration of normal muscle dynamics also improves myocardial energetics, underscoring mavacamten's therapeutic potential in managing HCM. The transitions between these states accentuate the impact of HCM on sarcomere function and mavacamten's corrective effects, emphasizing its role in rebalancing cardiac muscle performance [163, 164, 175].

Meanwhile, Aficamten has been found to decrease the activity of myosin ATPase in bovine cardiac myofibrils and reduce contractility in rat primary cardiomyocytes [177]. While its impact on the super-relaxed (SRX) state of myosin remains unknown, aficamten has been shown to interact with the ATP-binding site of myosin [177], suggesting a distinct mechanism of action. Table 6 shows a comparative analysis for sarcomeric contractile inhibitors in HCM.

**Table 6:** Summarizes the mechanisms of action, clinical trial outcomes, and efficacy of sarcomeric contractile inhibitors, including mavacamten and aficamten, in treating HCM

Type	Drug	Target	Mechanism	Contractility	Clinical Trials	Outcomes
Sarcomeric contractile inhibitors	Mavacamtem	Myosin heavy chain-β [171]	Stabilizes SRX state of myosin [169]	Reduced myosin head availability [163]	EXPLORER-HCM [146, 165, 188], PIONEER-HCM [170], MAVERICK-HCM [189, 190]	Improved peak oxygen consumption, NYHA class, reduced LVOT-obstruction [146, 165, 170, 188, 189, 190]
	Aficamten [178]	Myosin heavy chain-β [177]	Slows phosphate release from myosin [32]	Stabilizes weak actin-binding myosin [177]	REDWOOD-HCM [178]	Therapy led to significant symptom alleviation and biomarker improvement in over half of non-oHCM patients, with nearly a third achieving complete symptom resolution [178]

HCM: Hypertrophic Cardiomyopathy, LVOT: Left Ventricular Outflow Tract, NYHA: New York Heart Association, nHCM: non-obstructive Hypertrophic Cardiomyopathy SRX: super relaxed state. Clinical trials: EXPLORER-HCM, PIONEER-HCM, MAVERICK-HCM, REDWOOD-HCM

The three clinical trials previously discussed consistently demonstrate mavacamten's effectiveness and relative safety for oHCM patients already receiving standard treatments. This cardiac myosin inhibitor has demonstrated a comprehensive benefit profile, including alleviation of cardiac obstruction, enhanced cardiac function, and significant improvements in physical performance, overall well-being, and symptom management. Additionally, it has shown a broad improvement in various clinical metrics, patient-reported outcomes, and biomarkers. Notably, this cardiac myosin inhibitor has decreased the necessity for invasive procedures in severely symptomatic oHCM patients who were already receiving optimal medical treatment for 16–32 weeks. The PIONEER-HCM and EXPLORER-HCM trials utilized pharmacokinetic monitoring to tailor treatment and optimize outcomes. Mavacamten's impact on cardiac structure and function was further explored in sub-studies within EXPLORER-HCM. A CMR sub-study [146] revealed that mavacamten achieved a remarkable reduction in left ventricular mass index, maximum LV wall thickness, and left atrial volume index (LAVI) compared to placebo after 30 weeks, with sustained improvements at 96 weeks [191]. An echocardiographic sub-study [166] demonstrated that mavacamten led to a significantly higher rate of complete resolution of systolic anterior motion and mitral regurgitation (MR) compared to placebo, with strong correlations between serum NT-proBNP level reduction and echocardiographic parameters. These findings confirm mavacamten's ability to improve cardiac structure and function in patients with oHCM.

The medical research trial demonstrated that mavacamten treatment led to substantial enhancements in patient outcomes, including comprehensive well-being and total symptom burden. The KCCQ-OSS showed a mean increase from baseline to Week 30, with the mavacamten group exhibiting a notably higher rise (14.9) compared to the placebo group (5.4; *P* < 0.0001). Benefits were observed across all domains, including symptom severity, physical functioning, social functioning, and overall quality of life, although these gains were not maintained post-treatment. Additionally, an analysis employing the EQ-5D-5L index score and EQ-VAS revealed that patients receiving mavacamten experienced relatively significant advancements in both measures at Week 30 compared to placebo, indicating notable progress in health-related quality of life and overall well-being [192–194]. These clinical trial results demonstrate that mavacamten yields significant enhancements in patient well-being and quality of life for individuals with oHCM. Notably, the transition of 13 patients from the initial study to a long-term extension study paved the way for investigating the sustained safety and efficacy of cardiac myosin inhibitors in symptomatic oHCM [170]. Over a median follow-up of 201 weeks, mavacamten consistently demonstrated significant improvements, including substantial reductions in LVOT gradients and enhanced diastolic function metrics. Additionally, there were notable decreases in NT-proBNP levels, reflecting reduced myocardial wall stress [165, 166, 195]. These results highlight mavacamten's potential as a promising long-term treatment for obstructive HCM, supported by stable clinical outcomes and manageable safety profiles observed over extended monitoring periods [195].

The VALOR-HCM trial revealed a significant breakthrough in the treatment of symptomatic oHCM, demonstrating that mavacamten surpasses placebo in reducing high-sensitivity cardiac troponin I (hs‐cTnI) by 50% and NT‐proBNP by 80% (*P* < 0.01) [146]. This pioneering study also uncovered a crucial link between changes in left ventricular (LV) mass index, as assessed by CMR, and hs‐cTnI levels. Moreover, the VANISH trial's findings showed that early intervention with valsartan, an angiotensin receptor blocker, leads to improved cardiac structure, function, and remodeling outcomes, accompanied by stable or reduced NT‐proBNP levels over a 2-year period, compared to placebo [187]. Building on this success, the VALOR-HCM trial further demonstrated that mavacamten significantly reduces the need for SRT and improves NYHA functional class, outperforming placebo in both regards [147].

As far as clinical trials of aficamten are concerned, this phase 3 double-blind trial demonstrated a significant improvement in peak oxygen uptake among patients with symptomatic oHCM [32]. Aficamten, a novel oral agent targeting cardiac myosin, demonstrated efficacy in reducing LVOT gradients by modulating excessive cardiac contractility, resulting in improved exercise capacity and symptom alleviation. In a 24-week randomized controlled trial, 282 participants received either aficamten or placebo, with dose titration guided by serial echocardiographic assessments. This study design enabled a thorough evaluation of aficamten's safety and efficacy profile, providing valuable insights into its potential as a treatment for cardiac conditions characterized by hypercontractilit [32]. Aficamten significantly improved peak oxygen uptake (1.8 ml/kg/min vs. 0.0 ml/kg/min with placebo) and all 10 secondary endpoints, including quality of life, functional capacity, and pressure gradients [32]. Notably, the safety profile of aficamten was comparable to placebo, suggesting a favorable risk–benefit profile [32]. These findings support aficamten as a promising treatment option for patients with symptomatic obstructive HCM, offering improved exercise tolerance and reduced symptoms. As far as clinical trials of aficamten are concerned, this study provides robust evidence for its efficacy and safety, paving the way for further investigation into its long-term benefits and potential as a game-changer in HCM management.

For patients with refractory symptoms on initial therapy, mavacamten emerges as a pivotal secondary treatment option before considering surgical myectomy or SRT [1]. This innovative approach to refractory therapy leverages mavacamten's ability to target the underlying hypercontractility in HCM, offering a less invasive yet effective alternative to traditional surgical interventions. The efficacy of mavacamten in postponing or circumventing invasive procedures has been vividly demonstrated in trials such as VALOR-HCM [147]. This trial showcased mavacamten's capacity to substantially diminish the symptom burden, thus providing a valuable therapeutic window for patients who might otherwise face the prospect of surgery.While clinical trials have highlighted short-term side effects, the long-term effects, including impacts on lactation, pregnancy, and left ventricular ejection fraction (LVEF), warrant further investigation [175, 176]. The need for close surveillance and the expected high costs of mavacamten therapy create substantial practical and economic challenges [46, 175]. Furthermore, certain patient groups, including those with atrial fibrillation or advanced liver or kidney disease, were excluded from major trials, leaving uncertainties about mavacamten's safety in these populations [147, 165, 170]. As such, future research must address these gaps, particularly in diverse patient populations with coexisting conditions.

Mavacamten's therapeutic potential stretches beyond oHCM to include nHCM and diastolic heart failure,with current clinical trials investigating its effectiveness and safety in these additional indications [196]. Clinical trials of aficamten have demonstrated its promise in improving exercise tolerance and reducing symptoms in symptomatic oHCM patients [32], providing robust evidence for its efficacy and safety. These findings pave the way for further investigation into its long-term benefits and potential as a game-changer in HCM management, potentially leading to broader therapeutic uses and solidifying mavacamten's role as a versatile and innovative treatment in the cardiovascular domain.

## Conclusion

OHCM is a complex condition characterized by LVOT obstruction and impaired myocardial energetics, leading to significant morbidity and mortality. The findings of this research emphasize the need for a holistic strategy in addressing oHCM, bringing together genetic knowledge, innovative therapies, and tailored management plans.

The advent of novel therapeutics such as mavacamten and aficamten represents a significant milestone in the treatment landscape of oHCM. These myosin inhibitors have shown promising results in improving symptoms, functional capacity, and overall quality of life for patients. Mavacamten, in particular, has demonstrated efficacy in reducing the need for invasive septal reduction therapies, marking a paradigm shift in non-surgical intervention options.

This study highlights the critical impact of genetic diagnosis and informed decision-making in identifying at-risk individuals and tailoring personalized treatment plans. Emerging therapies, including other myosin inhibitors and adjunctive pharmacologic agents, continue to be explored, offering hope for more targeted and effective management of oHCM. The integration of genetic understanding with innovative therapies such as mavacamten and aficamten, alongside traditional management approaches, provides a comprehensive framework for optimizing outcomes in patients with oHCM. Continued research and clinical trials are essential to further refine these strategies and improve the prognosis for individuals affected by this challenging condition.

## Data Availability

Data available within the article. The authors confirm that the data supporting the findings of this study are available within the article.

## Abbreviations

HCM: Hypertrophic cardiomyopathy
oHCM: Obstructive hypertrophic cardiomyopathy
LVOT: Left ventricular outflow tract
LV: Left ventricle
NYHA: New York Heart Association
CMR: Cardiac magnetic resonance imaging
SRT: Septal reduction therapy
ESM: Extended septal myectomy
SCD: Sudden cardiac death
FDA: Food and drug administration

## References

[1] Ommen SR, Mital S, Burke MA, Day SM, Deswal A, Elliott P et al (2020) 2020 AHA/ACC guideline for the diagnosis and treatment of patients with hypertrophic cardiomyopathy: a report of the American College of Cardiology/American Heart Association Joint Committee on Clinical Practice Guidelines. J Am Coll Cardiol 76(25):e159–e240 33229116 10.1016/j.jacc.2020.08.045

[2] Marian AJ, Braunwald E (2017) Hypertrophic cardiomyopathy: genetics, pathogenesis, clinical manifestations, diagnosis, and therapy. Circ Res 121(7):749–770 28912181 10.1161/CIRCRESAHA.117.311059 PMC5654557

[3] Lehman SJ, Crocini C, Leinwand LA (2022) Targeting the sarcomere in inherited cardiomyopathies. Nat Rev Cardiol 19(6):353–363 35304599 10.1038/s41569-022-00682-0 PMC9119933

[4] Maron BJ, Gardin JM, Flack JM, Gidding SS, Kurosaki TT, Bild DE (1995) Prevalence of hypertrophic cardiomyopathy in a general population of young adults: echocardiographic analysis of 4111 subjects in the CARDIA study. Circulation 92(4):785–789 7641357 10.1161/01.cir.92.4.785

[5] Maron BJ, Ommen SR, Semsarian C, Spirito P, Olivotto I, Maron MS (2014) Hypertrophic cardiomyopathy: present and future, with translation into contemporary cardiovascular medicine. J Am Coll Cardiol 64(1):83–99 24998133 10.1016/j.jacc.2014.05.003

[6] Maron MS, Hellawell JL, Lucove JC, Farzaneh-Far R, Olivotto I (2016) Occurrence of clinically diagnosed hypertrophic cardiomyopathy in the United States. Am J Cardiol 117(10):1651–1654 27006153 10.1016/j.amjcard.2016.02.044

[7] Semsarian C, Ingles J, Maron MS, Maron BJ (2015) New perspectives on the prevalence of hypertrophic cardiomyopathy. J Am Coll Cardiol 65(12):1249–1254 25814232 10.1016/j.jacc.2015.01.019

[8] Mazzarotto F, Olivotto I, Boschi B, Girolami F, Poggesi C, Barton PJ et al (2020) Contemporary insights into the genetics of hypertrophic cardiomyopathy: toward a new era in clinical testing? J Am Heart Assoc 9(8):e015473 32306808 10.1161/JAHA.119.015473 PMC7428545

[9] Watkins H (2021) Time to think differently about sarcomere-negative hypertrophic cardiomyopathy. Circulation 143(25):2415–2417 34152793 10.1161/CIRCULATIONAHA.121.053527

[10] Maron MS, Olivotto I, Zenovich AG, Link MS, Pandian NG, Kuvin JT et al (2006) Hypertrophic cardiomyopathy is predominantly a disease of left ventricular outflow tract obstruction. Circulation 114(21):2232–2239 17088454 10.1161/CIRCULATIONAHA.106.644682

[11] Stewart S, Mason DT, Braunwald E (1968) Impaired rate of left ventricular filling in idiopathic hypertrophic subaortic stenosis and valvular aortic stenosis. Circulation 37(1):8–14 5688694 10.1161/01.cir.37.1.8

[12] Maron BJ, Rowin EJ, Maron MS (2019) Letter by Maron et al regarding article, “genotype and lifetime burden of disease in hypertrophic cardiomyopathy: insights from the sarcomeric human cardiomyopathy registry (SHaRe).” Circulation 139(12):1557–1558 30883223 10.1161/CIRCULATIONAHA.118.038189

[13] Capota R, Militaru S, Ionescu AA, Rosca M, Baicus C, Popescu BA et al (2020) Quality of life status determinants in hypertrophic cardiomyopathy as evaluated by the Kansas City Cardiomyopathy Questionnaire. Health Qual Life Outcomes 18:1–8 33126893 10.1186/s12955-020-01604-9 PMC7602300

[14] Guttmann OP, Rahman MS, O’Mahony C, Anastasakis A, Elliott PM (2014) Atrial fibrillation and thromboembolism in patients with hypertrophic cardiomyopathy: systematic review. Heart 100(6):465–472 24014282 10.1136/heartjnl-2013-304276

[15] Ho CY, Day SM, Ashley EA, Michels M, Pereira AC, Jacoby D et al (2018) Genotype and lifetime burden of disease in hypertrophic cardiomyopathy: insights from the Sarcomeric Human Cardiomyopathy Registry (SHaRe). Circulation 138(14):1387–1398 30297972 10.1161/CIRCULATIONAHA.117.033200 PMC6170149

[16] Marstrand P, Han L, Day SM, Olivotto I, Ashley EA, Michels M et al (2020) Hypertrophic cardiomyopathy with left ventricular systolic dysfunction: insights from the SHaRe registry. Circulation 141(17):1371–1383 32228044 10.1161/CIRCULATIONAHA.119.044366 PMC7182243

[17] Spirito P, Autore C, Rapezzi C, Bernabò P, Badagliacca R, Maron MS et al (2009) Syncope and risk of sudden death in hypertrophic cardiomyopathy. Circulation 119(13):1703–1710 19307481 10.1161/CIRCULATIONAHA.108.798314

[18] Maron BJ, Shen W-K, Link MS, Epstein AE, Almquist AK, Daubert JP et al (2000) Efficacy of implantable cardioverter–defibrillators for the prevention of sudden death in patients with hypertrophic cardiomyopathy. N Engl J Med 342(6):365–373 10666426 10.1056/NEJM200002103420601

[19] Elliott PM, Anastasakis A, Borger MA, Borggrefe M, Cecchi F, Charron P et al (2014) 2014 ESC guidelines on diagnosis and management of hypertrophic cardiomyopathy. Polish Heart J (Kardiologia Polska) 72(11):1054–1126 10.5603/KP.2014.0212 25524376

[20] Members WC, Ommen SR, Ho CY, Asif IM, Balaji S, Burke MA et al (2024) 2024 AHA/ACC/AMSSM/HRS/PACES/SCMR guideline for the management of hypertrophic cardiomyopathy: a report of the American Heart Association/American College of Cardiology Joint Committee on Clinical Practice Guidelines. J Am Coll Cardiol 83(23):2324–2405 38727647 10.1016/j.jacc.2024.02.014

[21] Maron BJ, Dearani JA, Ommen SR, Maron MS, Schaff HV, Nishimura RA et al (2015) Low operative mortality achieved with surgical septal myectomy: role of dedicated hypertrophic cardiomyopathy centers in the management of dynamic subaortic obstruction. J Am Coll Cardiol 66(11):1307–1308 26361164 10.1016/j.jacc.2015.06.1333

[22] Hodges K, Rivas CG, Aguilera J, Borden R, Alashi A, Blackstone EH et al (2019) Surgical management of left ventricular outflow tract obstruction in a specialized hypertrophic obstructive cardiomyopathy center. J Thorac Cardiovasc Surg 157(6):2289–2299 30782406 10.1016/j.jtcvs.2018.11.148

[23] Hong JH, Schaff HV, Nishimura RA, Abel MD, Dearani JA, Li Z et al (2016) Mitral regurgitation in patients with hypertrophic obstructive cardiomyopathy: implications for concomitant valve procedures. J Am Coll Cardiol 68(14):1497–1504 27687190 10.1016/j.jacc.2016.07.735

[24] Nguyen A, Schaff HV, Nishimura RA, Dearani JA, Geske JB, Lahr BD et al (2018) Does septal thickness influence outcome of myectomy for hypertrophic obstructive cardiomyopathy? Eur J Cardiothorac Surg 53(3):582–589 29182736 10.1093/ejcts/ezx398

[25] Balaram SK, Ross RE, Sherrid MV, Schwartz GS, Hillel Z, Winson G et al (2012) Role of mitral valve plication in the surgical management of hypertrophic cardiomyopathy. Ann Thorac Surg 94(6):1990–1998 22858269 10.1016/j.athoracsur.2012.06.008

[26] Rastegar H, Boll G, Rowin EJ, Dolan N, Carroll C, Udelson JE et al (2017) Results of surgical septal myectomy for obstructive hypertrophic cardiomyopathy: the Tufts experience. Ann Cardiothorac Surg 6(4):353 28944176 10.21037/acs.2017.07.07 PMC5602194

[27] Vriesendorp PA, Schinkel AF, Soliman OI, Kofflard MJ, de Jong PL, van Herwerden LA et al (2015) Long-term benefit of myectomy and anterior mitral leaflet extension in obstructive hypertrophic cardiomyopathy. Am J Cardiol 115(5):670–675 25591899 10.1016/j.amjcard.2014.12.017

[28] Ferrazzi P, Spirito P, Iacovoni A, Calabrese A, Migliorati K, Simon C et al (2015) Transaortic chordal cutting: mitral valve repair for obstructive hypertrophic cardiomyopathy with mild septal hypertrophy. J Am Coll Cardiol 66(15):1687–1696 26449139 10.1016/j.jacc.2015.07.069

[29] Minakata K, Dearani JA, Nishimura RA, Maron BJ, Danielson GK (2004) Extended septal myectomy for hypertrophic obstructive cardiomyopathy with anomalous mitral papillary muscles or chordae. J Thorac Cardiovasc Surg 127(2):481–489 14762358 10.1016/j.jtcvs.2003.09.040

[30] Kaple RK, Murphy RT, DiPaola LM, Houghtaling PL, Lever HM, Lytle BW et al (2008) Mitral valve abnormalities in hypertrophic cardiomyopathy: echocardiographic features and surgical outcomes. Ann Thorac Surg 85(5):1527–35.e2 18442532 10.1016/j.athoracsur.2008.01.061

[31] Schoendube FA, Klues HG, Reith S, Flachskampf FA, Hanrath P, Messmer BJ (1995) Long-term clinical and echocardiographic follow-up after surgical correction of hypertrophic obstructive cardiomyopathy with extended myectomy and reconstruction of the subvalvular mitral apparatus. Circulation 92(9):122–127 10.1161/01.cir.92.9.122 7586394

[32] Maron MS, Masri A, Nassif ME, Barriales-Villa R, Arad M, Cardim N et al (2024) Aficamten for symptomatic obstructive hypertrophic cardiomyopathy. N Engl J Med 390:1849–1861 38739079 10.1056/NEJMoa2401424

[33] Dorobantu LF, Iosifescu TA, Ticulescu R, Greavu M, Alexandrescu M, Dermengiu A et al (2022) Transaortic shallow septal myectomy and cutting of secondary fibrotic mitral valve chordae—a 5-year single-center experience in the treatment of hypertrophic obstructive cardiomyopathy. J Clin Med 11(11):3083 35683470 10.3390/jcm11113083 PMC9181673

[34] Rowin EJ, Sridharan A, Madias C, Firely C, Koethe B, Link MS et al (2020) Prediction and prevention of sudden death in young patients (< 20 years) with hypertrophic cardiomyopathy. Am J Cardiol 128:75–83 32650928 10.1016/j.amjcard.2020.04.042

[35] Chaowu Y, Shihua Z, Jian L, Li L, Wei F (2013) Cardiovascular magnetic resonance characteristics in children with hypertrophic cardiomyopathy. Circ Heart Fail 6(5):1013–1020 23873474 10.1161/CIRCHEARTFAILURE.113.000414

[36] Agarwal S, Tuzcu EM, Desai MY, Smedira N, Lever HM, Lytle BW et al (2010) Updated meta-analysis of septal alcohol ablation versus myectomy for hypertrophic cardiomyopathy. J Am Coll Cardiol 55(8):823–834 20170823 10.1016/j.jacc.2009.09.047

[37] Singh K, Qutub M, Carson K, Hibbert B, Glover C (2016) A meta analysis of current status of alcohol septal ablation and surgical myectomy for obstructive hypertrophic cardiomyopathy. Catheter Cardiovasc Interv 88(1):107–115 26526299 10.1002/ccd.26293

[38] Miron A, Lafreniere-Roula M, Steve Fan C-P, Armstrong KR, Dragulescu A, Papaz T et al (2020) A validated model for sudden cardiac death risk prediction in pediatric hypertrophic cardiomyopathy. Circulation 142(3):217–229 32418493 10.1161/CIRCULATIONAHA.120.047235 PMC7365676

[39] Norrish G, Ding T, Field E, McLeod K, Ilina M, Stuart G et al (2019) A validation study of the European Society of Cardiology guidelines for risk stratification of sudden cardiac death in childhood hypertrophic cardiomyopathy. EP Europace 21(10):1559–1565 31155643 10.1093/europace/euz118 PMC6788212

[40] Maurizi N, Passantino S, Spaziani G, Girolami F, Arretini A, Targetti M et al (2018) Long-term outcomes of pediatric-onset hypertrophic cardiomyopathy and age-specific risk factors for lethal arrhythmic events. JAMA Cardiol 3(6):520–525 29710196 10.1001/jamacardio.2018.0789 PMC6128509

[41] Balaji S, DiLorenzo MP, Fish FA, Etheridge SP, Aziz PF, Russell MW et al (2019) Risk factors for lethal arrhythmic events in children and adolescents with hypertrophic cardiomyopathy and an implantable defibrillator: an international multicenter study. Heart Rhythm 16(10):1462–1467 31026510 10.1016/j.hrthm.2019.04.040

[42] Norrish G, Cantarutti N, Pissaridou E, Ridout DA, Limongelli G, Elliott PM et al (2017) Risk factors for sudden cardiac death in childhood hypertrophic cardiomyopathy: a systematic review and meta-analysis. Eur J Prev Cardiol 24(11):1220–1230 28482693 10.1177/2047487317702519

[43] Maron BJ, Rowin EJ, Casey SA, Lesser JR, Garberich RF, McGriff DM et al (2016) Hypertrophic cardiomyopathy in children, adolescents, and young adults associated with low cardiovascular mortality with contemporary management strategies. Circulation 133(1):62–73 26518766 10.1161/CIRCULATIONAHA.115.017633

[44] Bharucha T, Lee KJ, Daubeney PE, Nugent AW, Turner C, Sholler GF et al (2015) Sudden death in childhood cardiomyopathy: results from a long-term national population-based study. J Am Coll Cardiol 65(21):2302–2310 26022819 10.1016/j.jacc.2015.03.552

[45] Mathew J, Zahavich L, Lafreniere-Roula M, Wilson J, George K, Benson L et al (2018) Utility of genetics for risk stratification in pediatric hypertrophic cardiomyopathy. Clin Genet 93(2):310–319 29053178 10.1111/cge.13157

[46] FD A (2022) Approved drug proucts: CAMZYOS (mavacamten) capsules for oral use

[47] Jackson G, Atkinson L, Oram S (1975) Double-blind comparison of tolamolol, propranolol, practolol, and placebo in the treatment of angina pectoris. Br Med J 1(5960):708–712 804952 10.1136/bmj.1.5960.708 PMC1672738

[48] Dybro A, Rasmussen T, Nielsen R, Andersen M, Jensen M, Poulsen S (2021) Clinical effects of metoprolol in obstructive hypertrophic cardiomyopathy (TEMPO). A randomized, double-blinded, placebo-controlled crossover trial. Eur Heart J 42:ehab724.1769

[49] Mooy J, van Baak M, Böhm R, Does R, Petri H, van Kemenade J et al (1987) The effects of verapamil and propranolol on exercise tolerance in hypertensive patients. Clin Pharmacol Ther 41(5):490–495 3552358 10.1038/clpt.1987.63

[50] Ulimoen SR, Enger S, Pripp AH, Abdelnoor M, Arnesen H, Gjesdal K et al (2014) Calcium channel blockers improve exercise capacity and reduce N-terminal Pro-B-type natriuretic peptide levels compared with beta-blockers in patients with permanent atrial fibrillation. Eur Heart J 35(8):517–524 24135831 10.1093/eurheartj/eht429

[51] Javidgonbadi D (2019) Factors in. uencing outcome in patients with obstructive hypertrophic cardiomyopathy

[52] Braunwald E, Saberi S, Abraham TP, Elliott PM, Olivotto I (2023) Mavacamten: a first-in-class myosin inhibitor for obstructive hypertrophic cardiomyopathy. Eur Heart J 44(44):4622–4633 37804245 10.1093/eurheartj/ehad637 PMC10659958

[53] Firth J (2019) Cardiology: hypertrophic cardiomyopathy. Clin Med 19(1):61–63 10.7861/clinmedicine.19-1-61 PMC6399630 30651247

[54] Pantazis A, Vischer AS, Perez-Tome MC, Castelletti S (2015) Diagnosis and management of hypertrophic cardiomyopathy. Echo Res Pract 2(1):R45–R53 26693331 10.1530/ERP-15-0007 PMC4676455

[55] Klues HG, Maron BJ, Dollar AL, Roberts WC (1992) Diversity of structural mitral valve alterations in hypertrophic cardiomyopathy. Circulation 85(5):1651–1660 1572023 10.1161/01.cir.85.5.1651

[56] Maron MS, Olivotto I, Betocchi S, Casey SA, Lesser JR, Losi MA et al (2003) Effect of left ventricular outflow tract obstruction on clinical outcome in hypertrophic cardiomyopathy. N Engl J Med 348(4):295–303 12540642 10.1056/NEJMoa021332

[57] O’Mahony C, Jichi F, Pavlou M, Monserrat L, Anastasakis A, Rapezzi C et al (2014) A novel clinical risk prediction model for sudden cardiac death in hypertrophic cardiomyopathy (HCM risk-SCD). Eur Heart J 35(30):2010–2020 24126876 10.1093/eurheartj/eht439

[58] Niimura H, Patton KK, McKenna WJ, Soults J, Maron BJ, Seidman J et al (2002) Sarcomere protein gene mutations in hypertrophic cardiomyopathy of the elderly. Circulation 105(4):446–451 11815426 10.1161/hc0402.102990

[59] Van Dijk SJ, Dooijes D, dos Remedios C, Michels M, Lamers JM, Winegrad S et al (2009) Cardiac myosin-binding protein C mutations and hypertrophic cardiomyopathy: haploinsufficiency, deranged phosphorylation, and cardiomyocyte dysfunction. Circulation 119(11):1473–1483 19273718 10.1161/CIRCULATIONAHA.108.838672

[60] Popp MW, Maquat LE (2016) Leveraging rules of nonsense-mediated mRNA decay for genome engineering and personalized medicine. Cell 165(6):1319–1322 27259145 10.1016/j.cell.2016.05.053 PMC4924582

[61] Siwaszek A, Ukleja M, Dziembowski A (2014) Proteins involved in the degradation of cytoplasmic mRNA in the major eukaryotic model systems. RNA Biol 11(9):1122–1136 25483043 10.4161/rna.34406 PMC4615280

[62] Chen SN, Czernuszewicz G, Tan Y, Lombardi R, Jin J, Willerson JT et al (2012) Human molecular genetic and functional studies identify TRIM63, encoding Muscle RING Finger Protein 1, as a novel gene for human hypertrophic cardiomyopathy. Circ Res 111(7):907–919 22821932 10.1161/CIRCRESAHA.112.270207 PMC3482312

[63] Li R-K, Li G, Mickle DA, Weisel RD, Merante F, Luss H et al (1997) Overexpression of transforming growth factor-β1 and insulin-like growth factor-I in patients with idiopathic hypertrophic cardiomyopathy. Circulation 96(3):874–881 9264495 10.1161/01.cir.96.3.874

[64] Helms AS, Alvarado FJ, Yob J, Tang VT, Pagani F, Russell MW et al (2016) Genotype-dependent and-independent calcium signaling dysregulation in human hypertrophic cardiomyopathy. Circulation 134(22):1738–1748 27688314 10.1161/CIRCULATIONAHA.115.020086 PMC5127749

[65] Ruggiero A, Chen SN, Lombardi R, Rodriguez G, Marian AJ (2013) Pathogenesis of hypertrophic cardiomyopathy caused by myozenin 2 mutations is independent of calcineurin activity. Cardiovasc Res 97(1):44–54 22987565 10.1093/cvr/cvs294 PMC3527764

[66] Fatkin D, McConnell BK, Mudd JO, Semsarian C, Moskowitz IG, Schoen FJ et al (2000) An abnormal Ca 2+ response in mutant sarcomere protein–mediated familial hypertrophic cardiomyopathy. J Clin Investig 106(11):1351–1359 11104788 10.1172/JCI11093 PMC381468

[67] Teekakirikul P, Eminaga S, Toka O, Alcalai R, Wang L, Wakimoto H et al (2010) Cardiac fibrosis in mice with hypertrophic cardiomyopathy is mediated by non-myocyte proliferation and requires Tgf-β. J Clin Investig 120(10):3520–3529 20811150 10.1172/JCI42028 PMC2947222

[68] Senthil V, Chen SN, Tsybouleva N, Halder T, Nagueh SF, Willerson JT et al (2005) Prevention of cardiac hypertrophy by atorvastatin in a transgenic rabbit model of human hypertrophic cardiomyopathy. Circ Res 97(3):285–292 16020756 10.1161/01.RES.0000177090.07296.ac PMC1201449

[69] Patel R, Nagueh SF, Tsybouleva N, Abdellatif M, Lutucuta S, Kopelen HA et al (2001) Simvastatin induces regression of cardiac hypertrophy and fibrosis and improves cardiac function in a transgenic rabbit model of human hypertrophic cardiomyopathy. Circulation 104(3):317–324 11457751 10.1161/hc2801.094031 PMC2768618

[70] Kuster DW, Mulders J, Ten Cate FJ, Michels M, Dos Remedios CG, da Costa Martins PA et al (2013) MicroRNA transcriptome profiling in cardiac tissue of hypertrophic cardiomyopathy patients with MYBPC3 mutations. J Mol Cell Cardiol 65:59–66 24083979 10.1016/j.yjmcc.2013.09.012

[71] Leptidis S, El Azzouzi H, Lok SI, de Weger R, Olieslagers S, Kisters N et al (2013) A deep sequencing approach to uncover the miRNOME in the human heart. PLoS ONE 8(2):e57800 23460909 10.1371/journal.pone.0057800 PMC3583901

[72] Yang W, Li Y, He F, Wu H (2015) Microarray profiling of long non-coding RNA (lncRNA) associated with hypertrophic cardiomyopathy. BMC Cardiovasc Disord 15:1–9 26141701 10.1186/s12872-015-0056-7 PMC4490660

[73] Marian A (2000) Pathogenesis of diverse clinical and pathological phenotypes in hypertrophic cardiomyopathy. Lancet 355(9197):58–60 10615904 10.1016/s0140-6736(99)06187-5

[74] Lan F, Lee AS, Liang P, Sanchez-Freire V, Nguyen PK, Wang L et al (2013) Abnormal calcium handling properties underlie familial hypertrophic cardiomyopathy pathology in patient-specific induced pluripotent stem cells. Cell Stem Cell 12(1):101–113 23290139 10.1016/j.stem.2012.10.010 PMC3638033

[75] Geisterfer-Lowrance AA, Christe M, Conner DA, Ingwall JS, Schoen FJ, Seidman CE et al (1996) A mouse model of familial hypertrophic cardiomyopathy. Science 272(5262):731–734 8614836 10.1126/science.272.5262.731

[76] Tardiff JC, Factor SM, Tompkins BD, Hewett TE, Palmer BM, Moore RL et al (1998) A truncated cardiac troponin T molecule in transgenic mice suggests multiple cellular mechanisms for familial hypertrophic cardiomyopathy. J Clin Investig 101(12):2800–2811 9637714 10.1172/JCI2389 PMC508871

[77] Frey N, Brixius K, Schwinger RH, Benis T, Karpowski A, Lorenzen HP et al (2006) Alterations of tension-dependent ATP utilization in a transgenic rat model of hypertrophic cardiomyopathy. J Biol Chem 281(40):29575–29582 16882671 10.1074/jbc.M507740200

[78] Oberst L, Zhao G, Park JT, Brugada R, Michael LH, Entman ML et al (1998) Dominant-negative effect of a mutant cardiac troponin T on cardiac structure and function in transgenic mice. J Clin Investig 102(8):1498–1505 9788962 10.1172/JCI4088 PMC508999

[79] Marian AJ, Wu Y, Lim D-S, McCluggage M, Youker K, Yu Q-t et al (1999) A transgenic rabbit model for human hypertrophic cardiomyopathy. J Clin Investig 104(12):1683–1692 10606622 10.1172/JCI7956 PMC409884

[80] Lim D-S, Oberst L, McCluggage M, Youker K, Lacy J, DeMayo F et al (2000) Decreased left ventricular ejection fraction in transgenic mice expressing mutant cardiac troponin T-Q92, responsible for human hypertrophic cardiomyopathy. J Mol Cell Cardiol 32(3):365–374 10731436 10.1006/jmcc.1999.1081

[81] Fraysse B, Weinberger F, Bardswell SC, Cuello F, Vignier N, Geertz B et al (2012) Increased myofilament Ca2+ sensitivity and diastolic dysfunction as early consequences of Mybpc3 mutation in heterozygous knock-in mice. J Mol Cell Cardiol 52(6):1299–1307 22465693 10.1016/j.yjmcc.2012.03.009 PMC3370652

[82] Debold EP, Schmitt JP, Patlak J, Beck S, Moore J, Seidman JG et al (2007) Hypertrophic and dilated cardiomyopathy mutations differentially affect the molecular force generation of mouse α-cardiac myosin in the laser trap assay. Am J Physiol Heart Circ Physiol 293(1):H284–H291 17351073 10.1152/ajpheart.00128.2007

[83] Lombardi R, Bell A, Senthil V, Sidhu J, Noseda M, Roberts R et al (2008) Differential interactions of thin filament proteins in two cardiac troponin T mouse models of hypertrophic and dilated cardiomyopathies. Cardiovasc Res 79(1):109–117 18349139 10.1093/cvr/cvn078 PMC2773799

[84] Solaro RJ, Varghese J, Marian A, Chandra M (2002) Molecular mechanisms of cardiac myofilament activation: modulation by pH and a troponin T mutant R92Q. Basic Res Cardiol 97:I102–I110 12479243 10.1007/s003950200038

[85] Gupte TM, Haque F, Gangadharan B, Sunitha MS, Mukherjee S, Anandhan S et al (2015) Mechanistic heterogeneity in contractile properties of α-tropomyosin (TPM1) mutants associated with inherited cardiomyopathies. J Biol Chem 290(11):7003–7015 25548289 10.1074/jbc.M114.596676 PMC4358124

[86] Kraft T, Witjas-Paalberends ER, Boontje NM, Tripathi S, Brandis A, Montag J et al (2013) Familial hypertrophic cardiomyopathy: functional effects of myosin mutation R723G in cardiomyocytes. J Mol Cell Cardiol 57:13–22 23318932 10.1016/j.yjmcc.2013.01.001

[87] Helms AS, Davis FM, Coleman D, Bartolone SN, Glazier AA, Pagani F et al (2014) Sarcomere mutation-specific expression patterns in human hypertrophic cardiomyopathy. Circ Cardiovasc Genet 7(4):434–443 25031304 10.1161/CIRCGENETICS.113.000448 PMC4254656

[88] Witjas-Paalberends ER, Piroddi N, Stam K, Van Dijk SJ, Oliviera VS, Ferrara C et al (2013) Mutations in MYH7 reduce the force generating capacity of sarcomeres in human familial hypertrophic cardiomyopathy. Cardiovasc Res 99(3):432–441 23674513 10.1093/cvr/cvt119

[89] Witjas-Paalberends ER, Güçlü A, Germans T, Knaapen P, Harms HJ, Vermeer AM et al (2014) Gene-specific increase in the energetic cost of contraction in hypertrophic cardiomyopathy caused by thick filament mutations. Cardiovasc Res 103(2):248–257 24835277 10.1093/cvr/cvu127

[90] Bloemink M, Deacon J, Langer S, Vera C, Combs A, Leinwand L et al (2014) The hypertrophic cardiomyopathy myosin mutation R453C alters ATP binding and hydrolysis of human cardiac β-myosin. J Biol Chem 289(8):5158–5167 24344137 10.1074/jbc.M113.511204 PMC3931073

[91] Nagueh SF, Chen S, Patel R, Tsybouleva N, Lutucuta S, Kopelen HA et al (2004) Evolution of expression of cardiac phenotypes over a 4-year period in the β-myosin heavy chain-Q403 transgenic rabbit model of human hypertrophic cardiomyopathy. J Mol Cell Cardiol 36(5):663–673 15135661 10.1016/j.yjmcc.2004.02.010 PMC2768620

[92] Lompre A, Nadal-Ginard B, Mahdavi V (1984) Expression of the cardiac ventricular alpha-and beta-myosin heavy chain genes is developmentally and hormonally regulated. J Biol Chem 259(10):6437–6446 6327679

[93] Miyata S, Minobe W, Bristow MR, Leinwand LA (2000) Myosin heavy chain isoform expression in the failing and nonfailing human heart. Circ Res 86(4):386–390 10700442 10.1161/01.res.86.4.386

[94] Reiser PJ, Portman MA, Ning X-H, Moravec CS (2001) Human cardiac myosin heavy chain isoforms in fetal and failing adult atria and ventricles. Am J Physiol Heart Circ Physiol 280(4):H1814–H1820 11247796 10.1152/ajpheart.2001.280.4.H1814

[95] Millat G, Bouvagnet P, Chevalier P, Dauphin C, Jouk PS, Da Costa A et al (2010) Prevalence and spectrum of mutations in a cohort of 192 unrelated patients with hypertrophic cardiomyopathy. Eur J Med Genet 53(5):261–267 20624503 10.1016/j.ejmg.2010.07.007

[96] Erdmann J, Daehmlow S, Wischke S, Senyuva M, Werner U, Raible J et al (2003) Mutation spectrum in a large cohort of unrelated consecutive patients with hypertrophic cardiomyopathy. Clin Genet 64(4):339–349 12974739 10.1034/j.1399-0004.2003.00151.x

[97] Richard P, Charron P, Carrier L, Ledeuil C, Cheav T, Pichereau C et al (2003) Hypertrophic cardiomyopathy: distribution of disease genes, spectrum of mutations, and implications for a molecular diagnosis strategy. Circulation 107(17):2227–2232 12707239 10.1161/01.CIR.0000066323.15244.54

[98] Marian A, Yu Q, Mares A, Hill R, Roberts R, Perryman M (1992) Detection of a new mutation in the beta-myosin heavy chain gene in an individual with hypertrophic cardiomyopathy. J Clin Investig 90(6):2156–2165 1361491 10.1172/JCI116101 PMC443366

[99] Watkins H, Seidman CE, Seidman J, Feng H, Sweeney HL (1996) Expression and functional assessment of a truncated cardiac troponin T that causes hypertrophic cardiomyopathy. Evidence for a dominant negative action. J Clin Investig 98(11):2456–2461 8958207 10.1172/JCI119063 PMC507702

[100] Saltzman AJ, Mancini-DiNardo D, Li C, Chung WK, Ho CY, Hurst S et al (2010) The cardiac myosin binding protein C Arg502Trp mutation: a common cause of hypertrophic cardiomyopathy. Circ Res 106(9):1549–1552 20378854 10.1161/CIRCRESAHA.109.216291 PMC2893345

[101] Page SP, Kounas S, Syrris P, Christiansen M, Frank-Hansen R, Andersen PS et al (2012) Cardiac myosin binding protein-C mutations in families with hypertrophic cardiomyopathy: disease expression in relation to age gender and long term outcome. Circ Cardiovasc Genet 5(2):156–166 22267749 10.1161/CIRCGENETICS.111.960831

[102] Alfares AA, Kelly MA, McDermott G, Funke BH, Lebo MS, Baxter SB et al (2015) Results of clinical genetic testing of 2,912 probands with hypertrophic cardiomyopathy: expanded panels offer limited additional sensitivity. Genet Med 17(11):880–888 25611685 10.1038/gim.2014.205

[103] Hodatsu A, Konno T, Hayashi K, Funada A, Fujita T, Nagata Y et al (2014) Compound heterozygosity deteriorates phenotypes of hypertrophic cardiomyopathy with founder MYBPC3 mutation: evidence from patients and zebrafish models. Am J Physiol Heart Circ Physiol 307(11):H1594–H1604 25281569 10.1152/ajpheart.00637.2013

[104] Dausse E, Komajda M, Fetler L, Dubourg O, Dufour C, Carrier L et al (1993) Familial hypertrophic cardiomyopathy. Microsatellite haplotyping and identification of a hot spot for mutations in the beta-myosin heavy chain gene. J Clin Investig 92(6):2807–2813 8254035 10.1172/JCI116900 PMC288481

[105] Coviello DA, Maron BJ, Spirito P, Watkins H, Vosberg H-P, Thierfelder L et al (1997) Clinical features of hypertrophic cardiomyopathy caused by mutation of a “hot spot” in the alpha-tropomyosin gene. J Am Coll Cardiol 29(3):635–640 9060904 10.1016/s0735-1097(96)00538-4

[106] Forissier JF, Carrier L, Farza H, Bonne G, Bercovici J, Richard P et al (1996) Codon 102 of the cardiac troponin T gene is a putative hot spot for mutations in familial hypertrophic cardiomyopathy. Circulation 94(12):3069–3073 8989109 10.1161/01.cir.94.12.3069

[107] Bos JM, Will ML, Gersh BJ, Kruisselbrink TM, Ommen SR, Ackerman MJ (eds) (2014) Characterization of a phenotype-based genetic test prediction score for unrelated patients with hypertrophic cardiomyopathy. In: Mayo clinic proceedings. Elsevier, Amsterdam 10.1016/j.mayocp.2014.01.025 PMC4234122 24793961

[108] Walsh R, Thomson KL, Ware JS, Funke BH, Woodley J, McGuire KJ et al (2017) Reassessment of Mendelian gene pathogenicity using 7,855 cardiomyopathy cases and 60,706 reference samples. Genet Med 19(2):192–203 27532257 10.1038/gim.2016.90 PMC5116235

[109] Walsh R, Buchan R, Wilk A, John S, Felkin LE, Thomson KL et al (2017) Defining the genetic architecture of hypertrophic cardiomyopathy: re-evaluating the role of non-sarcomeric genes. Eur Heart J 38(46):3461–3468 28082330 10.1093/eurheartj/ehw603 PMC5837460

[110] Ayça B, Sahin I, Kucuk SH, Akin F, Kafadar D, Avşar M et al (2015) Increased transforming growth factor-β levels associated with cardiac adverse events in hypertrophic cardiomyopathy. Clin Cardiol 38(6):371–377 25973737 10.1002/clc.22404 PMC6711023

[111] Guo Y, Wu X, Zheng X, Lu J, Wang S, Huang X (2017) Usefulness of preoperative transforming growth factor-beta to predict new onset atrial fibrillation after surgical ventricular septal myectomy in patients with obstructive hypertrophic cardiomyopathy. Am J Cardiol 120(1):118–123 28483207 10.1016/j.amjcard.2017.03.252

[112] Jumaah S, Çelekli A, Sucu M (2018) The role of human urotensin-II in patients with hypertrophic cardiomyopathy. J Immunoassay Immunochem 39(2):150–162 28686108 10.1080/15321819.2017.1344130

[113] Zhu A, Bews H, Cheung D, Nagalingam RS, Mittal I, Goyal V et al (2020) Scleraxis as a prognostic marker of myocardial fibrosis in hypertrophic cardiomyopathy (SPARC) study. Can J Physiol Pharmacol 98(7):459–465 32027517 10.1139/cjpp-2019-0636

[114] Podzimkova J, Palecek T, Kuchynka P, Marek J, Danek B, Jachymova M et al (2019) Plasma osteopontin levels in patients with dilated and hypertrophic cardiomyopathy. Herz 44(4):347–353 29147972 10.1007/s00059-017-4645-3

[115] Yıldız SS, Sahin I, Cetinkal G, Aksan G, Kucuk SH, Keskin K et al (2018) Usefulness of serum omentin-1 levels for the prediction of adverse cardiac events in patients with hypertrophic cardiomyopathy. Med Princ Pract 27(2):107–114 29402833 10.1159/000487396 PMC5968372

[116] Kitaoka H, Kubo T, Baba Y, Yamasaki N, Matsumura Y, Furuno T et al (2012) Serum tenascin-C levels as a prognostic biomarker of heart failure events in patients with hypertrophic cardiomyopathy. J Cardiol 59(2):209–214 22218323 10.1016/j.jjcc.2011.11.008

[117] Cambronero F, Marín F, Roldan V, Hernandez-Romero D, Valdes M, Lip GY (2009) Biomarkers of pathophysiology in hypertrophic cardiomyopathy: implications for clinical management and prognosis. Eur Heart J 30(2):139–151 19136482 10.1093/eurheartj/ehn538

[118] Kubo T, Kitaoka H, Okawa M, Yamanaka S, Hirota T, Baba Y et al (2011) Combined measurements of cardiac troponin I and brain natriuretic peptide are useful for predicting adverse outcomes in hypertrophic cardiomyopathy. Circ J 75(4):919–926 21304210 10.1253/circj.cj-10-0782

[119] Geske JB, McKie PM, Ommen SR, Sorajja P (2013) B-type natriuretic peptide and survival in hypertrophic cardiomyopathy. J Am Coll Cardiol 61(24):2456–2460 23602778 10.1016/j.jacc.2013.04.004

[120] Yang H-J, Liu X, Qu C, Shi S-B, Liang J-J, Yang B (2018) Usefulness of red blood cell distribution width to predict heart failure hospitalization in patients with hypertrophic cardiomyopathy. Int Heart J 59(4):779–785 29877309 10.1536/ihj.17-507

[121] Oka K, Tsujino T, Nakao S, Lee-Kawabata M, Ezumi A, Masai M et al (2008) Symptomatic ventricular tachyarrhythmia is associated with delayed gadolinium enhancement in cardiac magnetic resonance imaging and with elevated plasma brain natriuretic peptide level in hypertrophic cardiomyopathy. J Cardiol 52(2):146–153 18922389 10.1016/j.jjcc.2008.07.003

[122] Song C, Wang S, Guo Y, Zheng X, Lu J, Fang X et al (2019) Preoperative NT-proBNP predicts midterm outcome after septal myectomy. J Am Heart Assoc 8(4):e011075 30760079 10.1161/JAHA.118.011075 PMC6405667

[123] Gawor M, Śpiewak M, Kubik A, Wróbel A, Lutyńska A, Marczak M et al (2018) Circulating biomarkers of hypertrophy and fibrosis in patients with hypertrophic cardiomyopathy assessed by cardiac magnetic resonance. Biomarkers 23(7):676–682 29737871 10.1080/1354750X.2018.1474261

[124] Neubauer S, Kolm P, Ho CY, Kwong RY, Desai MY, Dolman SF et al (2019) Distinct subgroups in hypertrophic cardiomyopathy in the NHLBI HCM registry. J Am Coll Cardiol 74(19):2333–2345 31699273 10.1016/j.jacc.2019.08.1057 PMC6905038

[125] Kawasaki T, Sakai C, Harimoto K, Yamano M, Miki S, Kamitani T (2013) Usefulness of high-sensitivity cardiac troponin T and brain natriuretic peptide as biomarkers of myocardial fibrosis in patients with hypertrophic cardiomyopathy. Am J Cardiol 112(6):867–872 23746480 10.1016/j.amjcard.2013.04.060

[126] Fernandes F, Arteaga-Fernandez E, de Oliveira AM, Buck P, Marsiglia JDC, Matsumoto A et al (2012) Plasma pro-B-type natriuretic peptide testing as a screening method for hypertrophic cardiomyopathy. J Cardiac Fail 18(7):564–568 10.1016/j.cardfail.2012.04.005 22748490

[127] Zhou Y, Yuan J, Wang Y, Qiao S (2019) Predictive values of apelin for myocardial fibrosis in hypertrophic cardiomyopathy. Int Heart J 60(3):648–655 31019180 10.1536/ihj.18-598

[128] McGorrian CM, Lyster S, Roy A, Tarrant H, Codd M, Doran P et al (2013) Use of a highly-sensitive cardiac troponin I assay in a screening population for hypertrophic cardiomyopathy: a case-referent study. BMC Cardiovasc Disord 13:1–9 24020864 10.1186/1471-2261-13-70 PMC3849957

[129] Kubo T, Ochi Y, Baba Y, Sugiura K, Takahashi A, Hirota T et al (2020) Elevation of high-sensitivity cardiac troponin T and left ventricular remodelling in hypertrophic cardiomyopathy. ESC Heart Fail 7(6):3593–3600 33047518 10.1002/ehf2.12852 PMC7754740

[130] Moreno V, Hernández-Romero D, Vilchez JA, García-Honrubia A, Cambronero F, Casas T et al (2010) Serum levels of high-sensitivity troponin T: a novel marker for cardiac remodeling in hypertrophic cardiomyopathy. J Cardiac Fail 16(12):950–956 10.1016/j.cardfail.2010.07.245 21111984

[131] Yang C, Qiao S, Song Y, Liu Y, Tang Y, Deng L et al (2019) Procollagen type I carboxy-terminal propeptide (PICP) and MMP-2 are potential biomarkers of myocardial fibrosis in patients with hypertrophic cardiomyopathy. Cardiovasc Pathol 43:107150 31639652 10.1016/j.carpath.2019.107150

[132] Shim CY, Ha J-W, Choi E-Y, Lee H-J, Moon S-H, Kim J-M et al (2009) Relationship between serum biochemical markers of myocardial fibrosis and diastolic function at rest and with exercise in hypertrophic cardiomyopathy. Korean Circ J 39(12):519–524 20049137 10.4070/kcj.2009.39.12.519 PMC2801459

[133] Ho CY, López B, Coelho-Filho OR, Lakdawala NK, Cirino AL, Jarolim P et al (2010) Myocardial fibrosis as an early manifestation of hypertrophic cardiomyopathy. N Engl J Med 363(6):552–563 20818890 10.1056/NEJMoa1002659 PMC3049917

[134] Emet S, Dadashov M, Sonsoz MR, Cakir MO, Yilmaz M, Elitok A et al (2018) Galectin-3: a novel biomarker predicts sudden cardiac death in hypertrophic cardiomyopathy. Am J Med Sci 356(6):537–543 30342718 10.1016/j.amjms.2018.08.013

[135] Gawor M, Śpiewak M, Janas J, Kożuch K, Wróbel A, Mazurkiewicz Ł et al (2017) The usefulness of sST2 and galectin-3 as novel biomarkers for better risk stratification in hypertrophic cardiomyopathy. Polish Heart J (Kardiologia Polska) 75(10):997–1004 10.5603/KP.a2017.0118 28612913

[136] Hu D-J, Xu J, Du W, Zhang J-X, Zhong M, Zhou Y-N (2016) Cardiac magnetic resonance and galectin-3 level as predictors of prognostic outcomes for non-ischemic cardiomyopathy patients. Int J Cardiovasc Imaging 32(12):1725–1733 27566192 10.1007/s10554-016-0958-1

[137] Tülüce SY, Tülüce K, Çil Z, Emren SV, Akyildiz ZI, Ergene O (2016) Galectin-3 levels in patients with hypertrophic cardiomyopathy and its relationship with left ventricular mass index and function. Anatol J Cardiol 16(5):344 26488381 10.5152/AnatolJCardiol.2015.6191 PMC5336784

[138] Unno K, Shibata R, Izawa H, Hirashiki A, Murase Y, Yamada T et al (2010) Adiponectin acts as a positive indicator of left ventricular diastolic dysfunction in patients with hypertrophic cardiomyopathy. Heart 96(5):357–361 19648128 10.1136/hrt.2009.172320

[139] Kitaoka H, Kubo T, Okawa M, Yamasaki N, Matsumura Y, Nishinaga M et al (2010) Plasma adiponectin levels and left ventricular remodeling in hypertrophic cardiomyopathy. Int Heart J 51(1):51–55 20145352 10.1536/ihj.51.51

[140] Sahin I, Gungor B, Ozkaynak B, Uzun F, Küçük SH, Avci II et al (2017) Higher copeptin levels are associated with worse outcome in patients with hypertrophic cardiomyopathy. Clin Cardiol 40(1):32–37 27768229 10.1002/clc.22602 PMC6490334

[141] Duan X, Liu R, Luo XL, Gao XJ, Hu FH, Guo C et al (2020) The relationship between β1-adrenergic and M2-muscarinic receptor autoantibodies and hypertrophic cardiomyopathy. Exp Physiol 105(3):522–530 31808213 10.1113/EP088263

[142] Ntelios D, Georgiou E, Alexouda S, Malousi A, Efthimiadis G, Tzimagiorgis G (2022) A critical approach for successful use of circulating microRNAs as biomarkers in cardiovascular diseases: the case of hypertrophic cardiomyopathy. Heart Fail Rev 27:281 33656618 10.1007/s10741-021-10084-y

[143] Blackshear JL, Kusumoto H, Safford RE, Wysokinska E, Thomas CS, Waldo OA et al (2016) Usefulness of von Willebrand factor activity indexes to predict therapeutic response in hypertrophic cardiomyopathy. Am J Cardiol 117(3):436–442 26705879 10.1016/j.amjcard.2015.11.016

[144] Le Tourneau T, Susen S, Caron C, Millaire A, Maréchaux S, Polge A-S et al (2008) Functional impairment of von Willebrand factor in hypertrophic cardiomyopathy: relation to rest and exercise obstruction. Circulation 118(15):1550–1557 18809794 10.1161/CIRCULATIONAHA.108.786681

[145] Cambronero F, Vilchez JA, García-Honrubia A, Ruiz-Espejo F, Moreno V, Hernández-Romero D et al (2010) Plasma levels of von Willebrand factor are increased in patients with hypertrophic cardiomyopathy. Thromb Res 126(1):e46–e50 20156645 10.1016/j.thromres.2010.01.010

[146] Saberi S, Cardim N, Yamani M, Schulz-Menger J, Li W, Florea V et al (2021) Mavacamten favorably impacts cardiac structure in obstructive hypertrophic cardiomyopathy: EXPLORER-HCM cardiac magnetic resonance substudy analysis. Circulation 143(6):606–608 33190524 10.1161/CIRCULATIONAHA.120.052359

[147] Desai MY, Owens A, Geske JB, Wolski K, Naidu SS, Smedira NG et al (2022) Myosin inhibition in patients with obstructive hypertrophic cardiomyopathy referred for septal reduction therapy. J Am Coll Cardiol 80(2):95–108 35798455 10.1016/j.jacc.2022.04.048

[148] Matthia EL, Setteducato ML, Elzeneini M, Vernace N, Salerno M, Kramer CM et al (2022) Circulating biomarkers in hypertrophic cardiomyopathy. J Am Heart Assoc 11(23):e027618 36382968 10.1161/JAHA.122.027618 PMC9851432

[149] Maron MS, Chan RH, Kapur NK, Jaffe IZ, McGraw AP, Kerur R et al (2018) Effect of spironolactone on myocardial fibrosis and other clinical variables in patients with hypertrophic cardiomyopathy. Am J Med 131(7):837–841 29604289 10.1016/j.amjmed.2018.02.025

[150] Axelsson A, Iversen K, Vejlstrup N, Ho C, Norsk J, Langhoff L et al (2015) Efficacy and safety of the angiotensin II receptor blocker losartan for hypertrophic cardiomyopathy: the INHERIT randomised, double-blind, placebo-controlled trial. Lancet Diabetes Endocrinol 3(2):123–131 25533774 10.1016/S2213-8587(14)70241-4

[151] Dybro AM, Rasmussen TB, Nielsen RR, Andersen MJ, Jensen MK, Poulsen SH (2021) Randomized trial of metoprolol in patients with obstructive hypertrophic cardiomyopathy. J Am Coll Cardiol 78(25):2505–2517 34915981 10.1016/j.jacc.2021.07.065

[152] Dybro AM, Rasmussen TB, Nielsen RR, Ladefoged BT, Andersen MJ, Jensen MK et al (2022) Effects of metoprolol on exercise hemodynamics in patients with obstructive hypertrophic cardiomyopathy. J Am Coll Cardiol 79(16):1565–1575 35450573 10.1016/j.jacc.2022.02.024

[153] Coppini R, Ferrantini C, Yao L, Fan P, Del Lungo M, Stillitano F et al (2013) Late sodium current inhibition reverses electromechanical dysfunction in human hypertrophic cardiomyopathy. Circulation 127(5):575–584 23271797 10.1161/CIRCULATIONAHA.112.134932

[154] Olivotto I, Camici PG, Merlini PA, Rapezzi C, Patten M, Climent V et al (2018) Efficacy of ranolazine in patients with symptomatic hypertrophic cardiomyopathy: the RESTYLE-HCM randomized, double-blind, placebo-controlled study. Circ Heart Fail 11(1):e004124 29321131 10.1161/CIRCHEARTFAILURE.117.004124

[155] Rowin EJ, Maron BJ, Carrick RT, Patel PP, Koethe B, Wells S et al (2020) Outcomes in patients with hypertrophic cardiomyopathy and left ventricular systolic dysfunction. J Am Coll Cardiol 75(24):3033–3043 32553256 10.1016/j.jacc.2020.04.045

[156] Mohite PN, Zych B, Banner NR, Simon AR (2014) Refractory heart failure dependent on short-term mechanical circulatory support: what next? Heart transplant or long-term ventricular assist device. Artif Organs 38(4):276–281 23981114 10.1111/aor.12157

[157] MacIntyre C, Lakdawala NK (2016) Management of atrial fibrillation in hypertrophic cardiomyopathy. Circulation 133(19):1901–1905 27166348 10.1161/CIRCULATIONAHA.115.015085

[158] Kim DS, Chu EL, Keamy-Minor EE, Paranjpe ID, Tang WL, O’Sullivan JW et al (2024) One-year real-world experience with mavacamten and its physiologic effects on obstructive hypertrophic cardiomyopathy. Front Cardiovasc Med 11:1429230 39314763 10.3389/fcvm.2024.1429230 PMC11417615

[159] Masri A, Lester SJ, Stendahl JC, Hegde SM, Sehnert AJ, Balaratnam G et al (2024) Long-term safety and efficacy of mavacamten in symptomatic obstructive hypertrophic cardiomyopathy: interim results of the PIONEER-OLE Study. J Am Heart Assoc 13(8):e030607 38591260 10.1161/JAHA.123.030607 PMC11262496

[160] Toepfer CN, Garfinkel AC, Venturini G, Wakimoto H, Repetti G, Alamo L et al (2020) Myosin sequestration regulates sarcomere function, cardiomyocyte energetics, and metabolism, informing the pathogenesis of hypertrophic cardiomyopathy. Circulation 141(10):828–842 31983222 10.1161/CIRCULATIONAHA.119.042339 PMC7077965

[161] Green EM, Wakimoto H, Anderson RL, Evanchik MJ, Gorham JM, Harrison BC et al (2016) A small-molecule inhibitor of sarcomere contractility suppresses hypertrophic cardiomyopathy in mice. Science 351(6273):617–621 26912705 10.1126/science.aad3456 PMC4784435

[162] Kawas RF, Anderson RL, Ingle SRB, Song Y, Sran AS, Rodriguez HM (2017) A small-molecule modulator of cardiac myosin acts on multiple stages of the myosin chemomechanical cycle. J Biol Chem 292(40):16571–16577 28808052 10.1074/jbc.M117.776815 PMC5633120

[163] Anderson RL, Trivedi DV, Sarkar SS, Henze M, Ma W, Gong H et al (2018) Deciphering the super relaxed state of human β-cardiac myosin and the mode of action of mavacamten from myosin molecules to muscle fibers. Proc Natl Acad Sci 115(35):E8143–E8152 30104387 10.1073/pnas.1809540115 PMC6126717

[164] Awinda PO, Watanabe M, Bishaw Y, Huckabee AM, Agonias KB, Kazmierczak K et al (2021) Mavacamten decreases maximal force and Ca2+ sensitivity in the N47K-myosin regulatory light chain mouse model of hypertrophic cardiomyopathy. Am J Physiol Heart Circ Physiol 320(2):H881–H890 33337957 10.1152/ajpheart.00345.2020 PMC8082789

[165] Olivotto I, Oreziak A, Barriales-Villa R, Abraham TP, Masri A, Garcia-Pavia P et al (2020) Mavacamten for treatment of symptomatic obstructive hypertrophic cardiomyopathy (EXPLORER-HCM): a randomised, double-blind, placebo-controlled, phase 3 trial. Lancet 396(10253):759–769 32871100 10.1016/S0140-6736(20)31792-X

[166] Hegde SM, Lester SJ, Solomon SD, Michels M, Elliott PM, Nagueh SF et al (2021) Effect of mavacamten on echocardiographic features in symptomatic patients with obstructive hypertrophic cardiomyopathy. J Am Coll Cardiol 78(25):2518–2532 34915982 10.1016/j.jacc.2021.09.1381

[167] Stern JA, Markova S, Ueda Y, Kim JB, Pascoe PJ, Evanchik MJ et al (2016) A small molecule inhibitor of sarcomere contractility acutely relieves left ventricular outflow tract obstruction in feline hypertrophic cardiomyopathy. PLoS ONE 11(12):e0168407 27973580 10.1371/journal.pone.0168407 PMC5156432

[168] Grillo MP, Erve JC, Dick R, Driscoll JP, Haste N, Markova S et al (2019) In vitro and in vivo pharmacokinetic characterization of mavacamten, a first-in-class small molecule allosteric modulator of beta cardiac myosin. Xenobiotica 49(6):718–733 30044681 10.1080/00498254.2018.1495856

[169] Perera V, Gretler D, Seroogy J, Chiang M, Palmisano M, Florea V (2022) Pharmacokinetic drug-drug interaction study of mavacamten with verapamil in healthy subjects. Clin Pharm Drug Dev 11:24–25 10.1002/cpdd.1332 37771180

[170] Heitner SB, Jacoby D, Lester SJ, Owens A, Wang A, Zhang D et al (2019) Mavacamten treatment for obstructive hypertrophic cardiomyopathy: a clinical trial. Ann Intern Med 170(11):741–748 31035291 10.7326/M18-3016

[171] Heitner SB, Lester S, Wang A, Hegde SM, Fang L, Balaratnam G et al (2019) Precision pharmacological treatment for obstructive hypertrophic cardiomyopathy with mavacamten: one-year results from PIONEER-OLE. Circulation 140:A13962-A

[172] Tison GH, Siontis KC, Abreau S, Attia Z, Agarwal P, Balasubramanyam A et al (2022) Assessment of disease status and treatment response with artificial intelligence—enhanced electrocardiography in obstructive hypertrophic cardiomyopathy. J Am Coll Cardiol 79(10):1032–1034 35272798 10.1016/j.jacc.2022.01.005 PMC10101773

[173] Wheeler MT, Jacoby D, Elliott PM, Saberi S, Hegde SM, Lakdawala NK et al (2023) Effect of beta-blocker therapy on the response to mavacamten in patients with symptomatic obstructive hypertrophic cardiomyopathy. Eur J Heart Fail 25(2):260–270 36404399 10.1002/ejhf.2737

[174] Wang A, Spertus JA, Wojdyla DM, Abraham TP, Nilles EK, Owens AT et al (2024) Mavacamten for obstructive hypertrophic cardiomyopathy with or without hypertension: post-hoc analysis of the EXPLORER-HCM trial. Heart Fail 12(3):567–579 10.1016/j.jchf.2023.07.030 37855754

[175] Dong T, Alencherry B, Ospina S, Desai MY (2023) Review of mavacamten for obstructive hypertrophic cardiomyopathy and future directions. Drug Des Devel Ther 17:1097–1106 37064432 10.2147/DDDT.S368590 PMC10094472

[176] Zhao X, Liu H, Tian W, Fang L, Yu M, Wu X et al (2023) Safety, tolerability, pharmacokinetics, and pharmacodynamics of single and multiple doses of aficamten in healthy Chinese participants: a randomized, double-blind, placebo-controlled, phase 1 study. Front Pharmacol 14:1227470 37680714 10.3389/fphar.2023.1227470 PMC10482267

[177] Chuang C, Collibee S, Ashcraft L, Wang W, Vander Wal M, Wang X et al (2021) Discovery of aficamten (CK-274), a next-generation cardiac myosin inhibitor for the treatment of hypertrophic cardiomyopathy. J Med Chem 64(19):14142–14152 34606259 10.1021/acs.jmedchem.1c01290

[178] Masri A, Sherrid MV, Abraham TP, Choudhury L, Garcia-Pavia P, Kramer CM et al (2024) Efficacy and safety of aficamten in symptomatic nonobstructive hypertrophic cardiomyopathy: results from the REDWOOD-HCM trial, Cohort 4. J Cardiac Fail 30:1439 10.1016/j.cardfail.2024.02.020 38493832

[179] Hwee DT, Hartman JJ, Wang J, Wu Y, Schaletzky J, Paliwal P et al (2019) Pharmacologic characterization of the cardiac myosin inhibitor, CK-3773274: a potential therapeutic approach for hypertrophic cardiomyopathy. Circ Res 125:A332

[180] Hartman JJ, Hwee DT, Wang J, Wu Y, Schaletzky J, Paliwal P et al (2020) Characterization of the cardiac myosin inhibitor CK-3773274: a potential therapeutic approach for hypertrophic cardiomyopathy. Biophys J 118(3):596a

[181] Hwee DT, Wu Y, Cremin P, Morgan BP, Malik FI, Chin ER (2019) The cardiac myosin inhibitor, CK-3773274, reduces contractility in the R403q mouse model of hypertrophic cardiomyopathy. Circ Res 125:A615

[182] Cremin P, Xu D, Zamora J, Cheung J, Leung K, Chuang G, et al., (eds) (2020) In vivo pharmacokinetic characterization of CK-3773274. A Novel Cardiac Myosin Inhibitor. In: Abstract presented at the 2020 American Association of pharmaceutical scientists meeting and exposition

[183] Maron MS, Masri A, Choudhury L, Olivotto I, Saberi S, Wang A et al (2023) Phase 2 study of aficamten in patients with obstructive hypertrophic cardiomyopathy. J Am Coll Cardiol 81(1):34–45 36599608 10.1016/j.jacc.2022.10.020

[184] Cohen LS, Braunwald E (1968) Chronic beta adrenergic receptor blockade in the treatment of idiopathic hypertrophic subaortic stenosis. Prog Cardiovasc Dis 11(3):211–221 4914002 10.1016/0033-0620(68)90011-x

[185] Maron BJ, Dearani JA, Smedira NG, Schaff HV, Wang S, Rastegar H et al (2022) Ventricular septal myectomy for obstructive hypertrophic cardiomyopathy (analysis spanning 60 years of practice): AJC expert panel. Am J Cardiol 180:124–139 35965115 10.1016/j.amjcard.2022.06.007

[186] Batzner A, Pfeiffer B, Neugebauer A, Aicha D, Blank C, Seggewiss H (2018) Survival after alcohol septal ablation in patients with hypertrophic obstructive cardiomyopathy. J Am Coll Cardiol 72(24):3087–3094 30545446 10.1016/j.jacc.2018.09.064

[187] Ho CY, Day SM, Axelsson A, Russell MW, Zahka K, Lever HM et al (2021) Valsartan in early-stage hypertrophic cardiomyopathy: a randomized phase 2 trial. Nat Med 27(10):1818–1824 34556856 10.1038/s41591-021-01505-4 PMC8666141

[188] Ho CY, Olivotto I, Jacoby D, Lester SJ, Roe M, Wang A et al (2020) Study design and rationale of EXPLORER-HCM: evaluation of mavacamten in adults with symptomatic obstructive hypertrophic cardiomyopathy. Circ Heart Fail 13(6):e006853 32498620 10.1161/CIRCHEARTFAILURE.120.006853

[189] Ho CY, Mealiffe ME, Bach RG, Bhattacharya M, Choudhury L, Edelberg JM et al (2020) Evaluation of mavacamten in symptomatic patients with nonobstructive hypertrophic cardiomyopathy. J Am Coll Cardiol 75(21):2649–2660 32466879 10.1016/j.jacc.2020.03.064

[190] Owens A, Sherrid MV, Wong TC, Bach RG, Wever-Pinzon O, Rigolli M et al (2021) Long-term efficacy and safety of mavacamten in patients with non-obstructive hypertrophic cardiomyopathy: interim results from the MAVERICK-LTE cohort of the MAVA-LTE study. Circulation 144(Suppl_1):A9685-A

[191] Saberi S, Kramer CM, Oreziak A, Masri A, Villa RB, Abraham TP et al (2023) 96-week cardiac magnetic resonance (CMR) results of treatment with mavacamten from the explorer cohort of the mava-long-term extension (LTE) study in patients (PTS) with obstructive hypertrophic cardiomyopathy (HCM). J Am Coll Cardiol 81(8):326

[192] Spertus JA, Fine JT, Elliott P, Ho CY, Olivotto I, Saberi S et al (2021) Mavacamten for treatment of symptomatic obstructive hypertrophic cardiomyopathy (EXPLORER-HCM): health status analysis of a randomised, double-blind, placebo-controlled, phase 3 trial. Lancet 397(10293):2467–2475 34004177 10.1016/S0140-6736(21)00763-7

[193] Nassif M, Fine JT, Dolan C, Reaney M, Addepalli P, Allen VD et al (2022) Validation of the Kansas City cardiomyopathy questionnaire in symptomatic obstructive hypertrophic cardiomyopathy. Heart Failure 10(8):531–539 35902155 10.1016/j.jchf.2022.03.002

[194] Xie J, Wang Y, Xu Y, Fine JT, Lam J, Garrison LP (2022) Assessing health-related quality-of-life in patients with symptomatic obstructive hypertrophic cardiomyopathy: EQ-5D-based utilities in the EXPLORER-HCM trial. J Med Econ 25(1):51–58 34907813 10.1080/13696998.2021.2011301

[195] Rader F, Oręziak A, Choudhury L, Saberi S, Fermin D, Wheeler MT et al (2024) Mavacamten treatment for symptomatic obstructive hypertrophic cardiomyopathy: interim results from the MAVA-LTE study, EXPLORER-LTE cohort. Heart Fail 12(1):164–177 10.1016/j.jchf.2023.09.028 38176782

[196] Dong T, Nissen S, Ospina S, Desai MY (2023) An evaluation of mavacamten for the treatment of symptomatic obstructive hypertrophic cardiomyopathy in adults. Expert Rev Cardiovasc Ther 21(1):5–13 36522857 10.1080/14779072.2023.2159811

